# Deciphering the Differential Effective and Toxic Responses of Bupleuri Radix following the Induction of Chronic Unpredictable Mild Stress and in Healthy Rats Based on Serum Metabolic Profiles

**DOI:** 10.3389/fphar.2017.00995

**Published:** 2018-01-15

**Authors:** Xiaoxia Gao, Meili Liang, Yuan Fang, Fang Zhao, Junsheng Tian, Xiang Zhang, Xuemei Qin

**Affiliations:** ^1^Modern Research Center for Traditional Chinese Medicine, Shanxi University, Taiyuan, China; ^2^Key Laboratory of Chemical Biology and Molecular Engineering of Ministry of Education, Shanxi University, Taiyuan, China; ^3^College of Chemistry and Chemical Engineering, Shanxi University, Taiyuan, China; ^4^Department of Chemistry, University of Louisville, Louisville, KY, United States

**Keywords:** bupleuri radix, anti-depression, toxic response, UPLC-MS, metabolic profiles

## Abstract

The petroleum ether fraction of Bupleuri Radix which is contained in the traditional Chinese medicine prescription of Xiaoyaosan (XYS) may have a therapeutic effect in depressed subjects based on the results of our previous study. It has been reported that Bupleuri Radix can cause liver toxicity following overdosing or long-term use. Therefore, this study aimed to decipher the differential effective and toxic responses of Bupleuri Radix in chronic unpredictable mild stress (CUMS) (with depression) and healthy rats based on serum metabolic profiles. Serum metabolic profiles were obtained using the UHPLC- Q Exactive Orbitrap-MS technique. Our results demonstrated that the petroleum ether fraction of Bupleuri Radix (PBR) produces an antidepressant effect through regulating glycometabolism, amino acid metabolism, sphingolipid metabolism, glycerophospholipid metabolism, and fatty acid metabolism. It also induces more severe toxic reactions in the liver or kidney in healthy rats than in CUMS rats, which exhibited a comparatively mild drug-induced toxic reaction. The altered lysine degradation, sphingolipid metabolism, glycerophospholipid metabolism, fatty acid metabolism, and bile acid metabolism could be at least partly responsible for the PBR toxic responses in healthy rats. The differential effective and toxic response of PBR in CUMS rats and healthy rats provide a new standard for the more rational and safer application of clinical drugs in the future.

## Introduction

Depression is a complex psychiatric illness leading to low morale, weight loss and anhedonia (Fabricatore and Wadden, 2006; Paykel, 2006) and ranked by the World Health Organization (WHO) as the third commonest cause of global disease burden (Mathers and Loncar, 2006). Synthetic chemical drugs have been used to treat depression, however, they have been found to produce severe side-effects such as psychomotor impairment and dependence (Sarko, 2000). Traditional Chinese medicine has been proven to have mild antidepressant benefits with fewer side-effects (Lu A. P. et al., 2012). Xiaoyaosan (XYS) is a traditional Chinese medicine prescription and has been widely used for centuries to treat depressive conditions. In our previous experiments, XYS was divided into five different polar fractions to explore the antidepressant activity (Zhou et al., 2011) and the results obtained showed that the petroleum ether fraction of XYS is the most effective fraction, suggesting that lipophilic components contribute to the antidepressant effect of XYS. In addition, previous studies in our laboratory have reported that there are 20 compounds in serum according to the theory of serum pharmacochemistry for the study of the petroleum ether fraction of XYS, and 13 of them are from Bupleuri Radix (Liu et al., 2013). These serum pharmacochemistry results show that Bupleuri Radix is the principal drug in the XYS prescription, which means that it plays a major therapeutic role among other drugs in the prescription. Nevertheless, the effects of only these compounds cannot explain all the actions of the petroleum ether fraction of Bupleuri Radix (PBR), and it is essential to see whether PBR has an anti-depressant effect.

Bupleuri Radix, the root of *Bupleurum chinense* DC. or *Bupleurum scorzonerifolium* Wild., is recorded as the highest grade of herbal drug in Shennong's Materia Medica (“Sheng Nong Ben Cao Jing” in Chinese), which is one of the earliest classic texts on traditional Chinese materia medica. Bupleuri Radix exhibits liver relief and hepatoprotective effects, and has been widely used to treat influenza, fever, malaria, hepatitis, jaundice, nephritis, dizziness, bitter taste in the mouth, lung diseases, cancer, and menstrual disorders (Yu, 1999; Cheng et al., 2012; Lu C. N. et al., 2012; Tzeng et al., 2012; Lei et al., 2017a,b). This herbal drug is frequently included in Chinese herbal formulas for the treatment of depression and is a major component of the prescription, such as XYS and Chaihu-Shu-Gan-San (Zhou et al., 2011; Su et al., 2014). Most of reports on Bupleuri Radix have suggested that it contains saponins, volatile oils, flavonoids and polysaccharides which have many pharmacological actions (Lei et al., 2017a; Yuan et al., 2017). Also, eight polyacetylene compounds have been isolated from Bupleuri Radix and have been found to inhibit monoamine neurotransmitter reuptake in studies we have carried out (Liu J. et al., 2015; Zhang et al., 2017). However, there have been no reports of the effect of polyacetylene compounds from Bupleuri Radix, except that bupleurotoxin and acetylbupleurotoxin from *Bupleurum longiradiatum* Turcz have a neurotoxic effect on the nervous system (Zhang et al., 2014). In addition, it has also been recorded that Bupleuri Radix can cause substantial damage to the liver if not used properly as described in ancient Chinese records. Modern reports show that Bupleuri Radix can cause acute liver injury, hepatocyte apoptosis and acute hepatitis if give in an overdose or on long-term treatment (Hsu et al., 2006; Yang et al., 2017). Therefore, it is crucial to investigate the actions and toxic responses of Bupleuri Radix in pathological and physiological conditions.

Metabolomics technologies provide a platform for discovering biomarkers and key pathways, based on global metabolite profiles in biological samples such as urine, plasma or tissue (Medina et al., 2014). They also provide diagnostic information and give a mechanistic insight into the biochemical effects of drugs (Medina et al., 2014). Currently, LC-MS is becoming the mainstream platform for metabolomics research due to its rapidity of analysis, high resolution and high sensitivity (Theodoridis et al., 2012; Forcisi et al., 2013; Zhao and Lin, 2014). Chronic unpredictable mild stress (CUMS) is an animal model of depression closely mimicing the human symptoms of depression (Willner, 1997). Venlafaxine hydrochloride is a novel serotonin-noradrenaline reuptake inhibitor (SNRI) which has been shown clinically to be an effective antidepressant with a faster onset of action than serotonin-specific reuptake inhibitors (SSRI) (Dawson et al., 1999). In this study, it was important to investigate the anti-depression effects of PBR in CUMS rats with venlafaxine hydrochloride as a positive control and the toxic response in healthy rats by using an LC-MS metabonomic approach to characterize the global serum metabolic profiling of rats.

## Materials and methods

### Chemicals and reagents

Petroleum ether (analytical grade) was obtained from Tianjin four Fine Chemicals Co. Ltd. (Tianjin, China). LC–MS grade acetonitrile and HPLC grade formic acid were obtained from Thermo-Fisher (USA) and the assay kits for ALT (P-1506011), AST (T-1504021), ALP (P-1502011), BUN (P-1411011), and CREA (B-1507091) were obtained from Shanghai Fuxing Changzheng Medical Science Co., Ltd. (Shanghai, China). The assay kit for TBA (160831) was obtained from Nanjing Jiancheng Bioengineering Institute (Nanjing, China). Standard reference samples of glucose monohydrate and galactose monohydrate were obtained from Tianjin Bodi Chemical Co., Ltd. (Tianjin, China). Glutamic acid, leucine, isoleucine, taurocholic acid sodium, sodium glycocholate hydrate and tryptophan were obtained from Sangon Biotech (Shanghai, China). Venlafaxine hydrochloride (Kang Hong Pharmaceutical, Chengdu, China, no. 071104) was obtained from Huang He drugstore (Taiyuan, China).

### Preparation of the petroleum ether fraction of Bupleuri Radix (PBR)

Bupleuri Radix (BR) was obtained from Shanxi Huayang Pharmaceutical Co., Ltd. and authenticated by Professor Xuemei Qin in the Pharmacognosy Department of Shanxi University. Bupleuri Radix (5 kg) was soaked in 95% ethanol for 12 h and then extracted (3 times) by thermal reflux for 2 h. After filtration, the ethanol extract was concentrated under reduced pressure and the residue was suspended in water and extracted 3 times with an equal volume of petroleum ether using an ultrasonic process (30 min for each ultrasonication). The petroleum ether extract was concentrated under reduced pressure and dried in a vacuum drying oven (60°C) to obtain the Petroleum Ether Fraction of Bupleuri Radix (PBR, about 150 g). The resulting residue was dissolved in water, containing 0.5% CMC-Na and 0.5% Tween-80, to give a series of extracts (D1, D2, D3, D4, D5, D6, and D7) with concentrations of 0.1, 0.3, 0.6, 1.25, 2.5, 5.0, and 10.0 g/ml (expressed as the weight of raw materials per ml). The main polyacetylene compounds of the PBR were detected by UPLC-PDA analysis (Zhang et al., 2017), which identified (2Z,8Z,10E)-pentadeca- 2,8,10-trien-4,6-diyn-ol (RB-1) 3.292 mg/g, (2Z,8E,10E)-pentadeca- 2,8,10-trien-4,6-diyn-ol (RB-2) 37.679 mg/g, (2Z,8Z,10E)-heptadeca- 2,8,10-trien-4,6-diyn-ol (RB-3) 1.723 mg/g, Bupleurynol (RB-4) 15.804 mg/g, (2Z,8E,10E)-pentadeca- 2,8,10-trien- 4,6-diyn-ylacetate (RB-5) 18.728 mg/g, (2E,8E,10E)-pentadeca- 2,8,10-trien- 4,6-diyn-yl acetate (RB-6) 10.043 mg/g, (2E,8E,10E)-pentadeca- 2,8,10-trien- 4,6-diyn-ol (RB-7) 10.586 mg/g, and (2E,8Z,10E)-heptadeca- 2,8,10-trien- 4,6-diyn-ol (RB-8) 5.364 mg/g (expressed as the weight of compound relative to PBR).

### Animals and their treatment

A total of 136 male Sprague-Dawley (SD) rats (200 ± 20 g, license no. SCXK (JING) 2012–0001) were obtained from the Experimental Animal Center of Beijing Weitong Lihua Technology Co. Ltd. They were fed under standard laboratory conditions and maintained on a 12 h light/dark cycle at a room temperature of 23–27°C and a humidity of 30–70%. Animals were allowed to adapt to the laboratory conditions for 1 week prior to the experiment. All animal experiments were performed under the NIH Guidelines for Care and Use of Laboratory Animals (U.S.A) and the Prevention of Cruelty to Animals Act (1986) of China and the experiments had also obtained approval from the Animal Ethics Committee of Shanxi University. The animals were randomly divided into 17 groups (*n* = 8): [K] healthy control rats, [Z1] ~ [Z7] healthy control rats given PBR at a concentration of D1 ~ D7, respectively; [CM] CUMS rats, [C1] ~ [C7] CUMS rats given PBR at a concentration of D1 ~ D7, respectively; [CY] CUMS rats given venlafaxine hydrochloride as a positive control. The CUMS procedure was performed as described previously (Tian et al., 2013). Rats in the [C1] ~ [C7], [CM], and [CY] groups were exposed to CUMS. The model rats were housed individually, while the rats in [Z1] ~ [Z7] and [K] groups were housed together. All the rats were given agents by gavage at a dose of 10 ml/kg body weight once daily for 21 days. Rats in the [K] and [CM] groups received an equal volume of vehicle orally. Rats in the [Z1] ~ [Z7] and [C1] ~ [C7] groups were received an equal volume of different concentrations of PBR of D1 ~ D7. Rats in the [CY] group received an equal volume of venlafaxine hydrochloride at a concentration of 0.0035 g/ml body weight.

### Behavior test

#### Body weight test

During the experiment, all the rats were weighed at 0, 7, 14, and 21 days and this index reflects the basic survival state of the rats.

#### Open-field test

The open-field test was performed as previously described (Tian et al., 2013) and was conducted in a quiet room (≤ 60 dB). Each animal was tested in an open-field apparatus which consisted of a square arena 100 × 100 cm, with a 70-cm-high side wall, and the floor was marked with a grid dividing it into 25 equal-sized squares. The test was performed at 0, 7, 14, and 21 days. Each rat was placed in the central square and observed for 5 min. Scores were calculated by the number of rearings (defined as standing upright on the hind legs), and the number of crossings (grid lines crossed by the rat with at least three paws).

#### Sucrose preference test

This was performed as described previously (Tian et al., 2013). After a 24-h period of water and food deprivation, each rat was placed in an individual metabolic cage in which two bottles containing water and 1% sucrose solution were placed at 0, 7, 14, and 21 days. The sucrose preference value was calculated as the percentage of 1% sucrose solution consumed relative to the total liquid intake within 4 h.

#### Statistical analysis

Quantitative data were presented as mean ± SEM. The significance of variations between groups in terms of behavior changes was determined using One-Way ANOVA with SPSS 16.0 software for Windows. Student's *T*-test was used to compare two groups and *P* < 0.05 was considered as statistically significant.

### Sample collection and biochemical index detection

Blood was collected from the femoral artery of the rats 1 h after administration on 21 days and centrifuged at 8,000 rpm for 10 min after standing for 0.5 h at 4°C. The serum was then transferred to fresh tubes and kept at −80°C for analysis. All the rats were sacrificed following blood collection. Liver and renal tissues were obtained and quickly frozen in liquid nitrogen and stored in −80°C until required for further investigation. A portion of the serum was used for routine biochemical index analysis of alanine aminotransferase (ALT), aspartate aminotransferase (AST), alkaline phosphatase (ALP), total bilirubin (TBIL), total bile acids (TBA), urea nitrogen (BUN), and creatinine (CREA) using an automatic biochemical analyzer (Konelab PRIME 7.2.1, Finland).

### Metabolomics analysis

The serum samples of rats in the [CM], [C4], [C6], [C7], [K], [Z4], [Z6], and [Z7] groups were used to obtain metabolic profiling data for metabolomics analysis. Samples (100 μL) of serum were added to 200 μL acetonitrile containing 0.1% formic acid, and the mixture was vortexed for 2 min. After being centrifuged at 13,000 rpm for 15 min at 4°C, each supernatant was collected in a UPLC vial for LC–MS analysis for metabolomics investigation.

### Metabolic profiling data acquisition

Metabolic profiling of serum samples based on untargeted analysis was collected using a Thermo-Fisher ultra-performance liquid chromatography system (UHPLC) (Thermo-Fisher, USA) coupled to a Q Exactive Orbitrap-MS (Thermo-Fisher, USA). Chromatographic separation was carried out on a Waters ACQUITYUPLC HSS T3 column (2.1 × 100 mm, 1.8 μm) maintained at 40°C. The mobile phase consisted of 0.1% formic acid in water (A) and 0.1% formic acid in acetonitrile (B), and operated under the following program: 0 ~ 2 min, 2% B; 2 ~ 3 min, 2 ~ 35% B; 3 ~ 17 min, 35 ~ 70% B, 17 ~ 18 min, 70% B; 18 ~ 29 min, 70 ~ 98% B; 29 ~ 31 min, 98% B; 31 ~ 33 min, 98 ~ 2% B; 33 ~ 35 min, 2% B. The sample injection volume was 5 μL and the flow rate was set at 0.2 ml/min.

The mass spectrometer was fitted with an electrospray ionization source and mass detection was carried out in both positive and negative ion modes with the following settings: capillary temperature, 320°C; heater temperature, 300°C; sheath gas velocity, 35 arb; auxiliary gas flow rate, 10 arb; scan range, m/z 100–1,500. Quality control (QC) samples were prepared by combining equal aliquots of plasma, processed in the same way as the analytical samples, and injected randomly throughout the run to monitor the stability of the LC/MS platform.

### Data processing and statistical analysis

The LC–MS raw data were exported using an Xcalibur workstation (Thermo Fisher Scientific Inc., Waltham, Ma, USA) and imported to Compound Discoverer 2.0 (Thermo Fisher, USA) to obtain the matched and aligned peak data. The processing parameters were as follows: mass range: 100–1,500 Da; mass tolerance: 5 ppm; RT tolerance [min]: 0.05; S/N threshold: 1.5. Then, the data were imported into Excel to carry out peak area normalization after being processed by Compound Discoverer 2.0.

SIMCA-P software (version 13.0, Umetrics, Sweden) was used for the principal components analysis (PCA), partial least-squares discriminant analysis (PLS-DA) and orthogonal partial least-squares (OPLS) analysis of the data from both positive and negative modes. The variable importance in the projection (VIP) value reflects the influence of every variable on the classification and the independent sample *t*-test was also included in the analysis of the discriminating variables. *P* < 0.05 and VIP > 1 were considered as statistically significant.

The database sources of the Human Metabolome Database (HMDB), KEGG and LIPID MAPS-Nature Lipidomics Gateway (http://www.lipidmaps.org/) and the related literature were queried with the exact masses of the metabolites to identify the differential metabolites and to better understand the metabolic pathways affected by the CUMS stress or PBR. The metabolite correlation network and metabolic networks were constructed using of Cytoscape software (v.3.5.0) (Zhou et al., 2016).

## Results

### Analysis of behavior test

#### Effects on body weight

**Figure 1:**
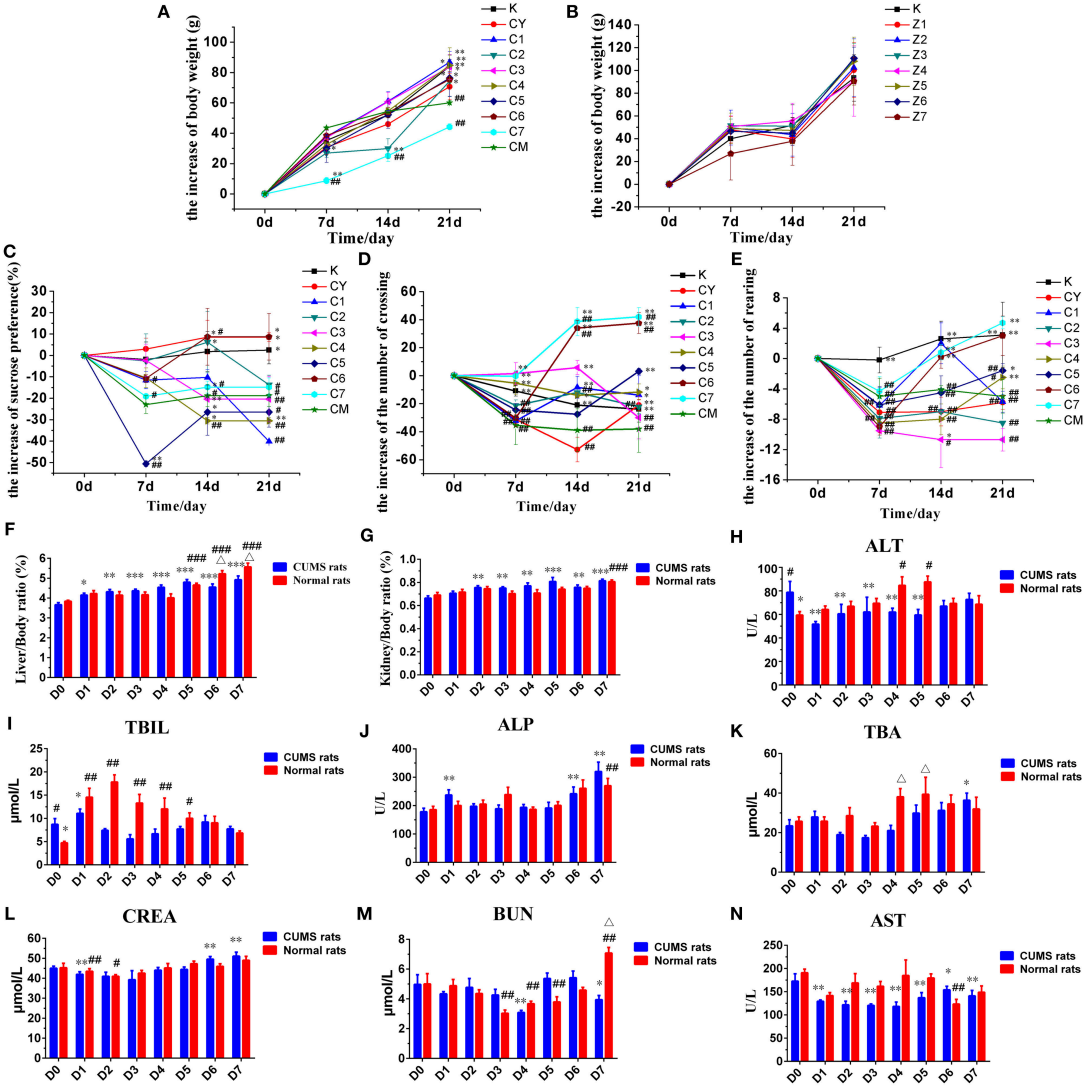
Effects of PBR on body weight of CUMS rats **(A)** and healthy rats **(B)**. Effects of PBR on the sucrose preference test **(C)**, the number of crossings **(D)** and the number of rearings **(E)**. Effects of PBR on biochemical parameters and visceral index: **(F)** Liver/Body weight ratio, **(G)** Kidney/Body weight ratio, **(H)** ALT, **(I)** TBIL, **(J)** ALP, **(K)** TBA, **(L)** CREA, **(M)** BUN, **(N)** AST. Data are presented as mean ± SEM. *n* = 8 each group. CUMS rats (◼blue), normal rats (◼red). D0: administration of equal volumes of vehicle. D1: the concentration of PBR was 0.1 g/ml (expressed as the weight of raw materials). D2: the concentration of PBR was 0.3 g/ml. D3: the concentration of PBR was 0.6 g/ml. D4: the concentration of PBR was 1.25 g/ml. D5: the concentration of PBR was 2.5 g/ml. D6: the concentration of PBR was 5.0 g/ml. D7: the concentration of PBR was 10.0 g/ml. ^*^P < 0.05, ^**^P < 0.01, ^***^P < 0.001 vs. [CM] group; ^#^*P* < 0.05, ^##^*P* < 0.01, ^###^*P* < 0.001 vs. [K] group; Δ*p* < 0.05, normal rats vs. CUMS rats at the same concentration of PBR in the biochemical parameters.

The weight of the rats was measured weekly during the stress period (0, 7, 14, and 21 days). There was no significant difference in the initial weight of rats in each group. The weight of rats in the [CM] group increased significantly slower than that in the [K] group after the end of CUMS stress period (*P* < 0.01, Figure 1A). The weight of rats in the [C1], [C2], [C3], [C4], [C5], and [C6] groups was significantly higher than that in the [CM] group at 21 days (*P* < 0.05, Figure 1A). However, the weight gain of rats in the [C7] group was significantly slower than in the [CM] group especially during the first 2 weeks (*P* < 0.01, Figure 1A), but without any significant difference in the 3rd week. The results obtained suggested that PBR can improve the slow body weight gain induced by CUMS stress, except for the high dose of PBR at D7 with transient injury.

The weights of the rats in [Z1] ~ [Z7] and [K] groups were all increased rapidly during the 3rd week (Figure 1B) and there was no significant difference in the weight of the rats in these groups compared with those in the [K] group.

#### Effects on sucrose preference test

The effects on the sucrose preference test in CUMS-induced rats was measured weekly (0, 7, 14, and 21 days) during the stress period. After 1 week, a different reduced degree of sucrose preference was seen but this was not statistically significant (Figure 1C). At 14 and 21 days, the sucrose preference was significantly reduced in the [CM] group compared with that in the [K] group (*P* < 0.05, Figure 1C), and the decline in sucrose consumption in the [C6] and [CY] groups was reversed (*P* < 0.05, Figure 1C). Furthermore, the decline in sucrose consumption in the [C6] group was reversed to a level close to that in the [K] group (*P* < 0.05, Figure 1C), and even exceeded the level in the [CY] group. However, the sucrose preference of [C4] group was significantly reduced compared with that in both the [CM] and [K] groups at 14 and 21 days (*P* < 0.01, Figure 1C). The sucrose preference in the [C1], [C2], [C3], [C5], and [C7] groups was reduced compared with that in the [CM] group at 21 days, but the difference was not statistically significant. These results implied that PBR could improve the anhedonia-like state at the concentration of D6 in the sucrose preference test.

#### Effects on open-field test

The effects on the open-field test in CUMS-induced rats was measured weekly (0, 7, 14, and 21 days) during the stress period. Results of the behavior in the open-field test revealed that the numbers of crossings and rearings were significantly lower in the [CM] group compared with those in the [K] group (*P* < 0.01, Figures 1D,E) starting on the seventh day after beginning CUMS. After 1 week, the numbers of crossings and rearings in all groups of CUMS-induced rats were reduced, however, they were increased in subsequent weeks with a different dose of PBR, except for those in the [CM] group. After 3 weeks, the numbers of both crossings and rearings in the [C4] ~ [C7] groups were significantly increased (*P* < 0.01 or *P* < 0.05, Figures 1D,E) compared with those in the [CM] group, and close to or exceeding the level in the [K] group, and even exceeded the level of [CY] in the [C5] ~ [C7] groups. However, those in the [C1], [C2], and [C3] groups showed no significant difference compared with those in the [CM] group, except for the numbers of crossings. These results indicated that PBR, at a dose of D4 ~ D7, could increase the locomotor activities in the CUMS-induced rats.

#### Effects on biochemical changes

The ratios of the weight of liver or kidney to body weight were analyzed (Figures 1F,G), when it was found that there was a slight reduction between the [CM] and [K] groups, but without any statistical significance. The ratios of the weight of liver to that of body weight were significantly increased in the [Z5] ~ [Z7] groups compared with those in the [K] group (*P* < 0.01, Figure 1F). In addition, there was a significant increase in CUMS-induced rats with different doses of PBR compared with those of rats in the [CM] group (*P* < 0.01, Figure 1F). However, the changes in the visceral index of liver showed that a high dose of D6 (*P* < 0.01) and D7 (*P* < 0.05) produced a higher up-regulation in healthy rats than in CUMS-induced rats (Figure 1F). The ratio of the weight of kidney to that of body weight was significantly increased only in the [Z7] group (*P* < 0.001, Figure 1G) compared with that in the [K] group. Also, the visceral index of kidney was comparatively mildly increased in CUMS rats with a different PBR dose, although it was significantly increased in the [C2] ~ [C7] groups compared with the [CM] group (*P* < 0.05, Figure 1G). These results suggested that a high dose of PBR might cause liver or kidney injury in healthy rats, but did have not much influence in CUMS-induced rats.

The effects on biochemical changes in rats was measured at 21 days. Compared with those in the [K] group, the levels of ALT and TBIL were significantly increased in the [CM] group (*P* < 0.05, Figures 1H,I), while the levels of the other indices, ALP, AST, TBA, CREA, and BUN, showed no significant changes in the [CM] group. The ALT level was significantly reduced in the [C1] ~ [C5] groups compared with that in the [CM] group (*P* < 0.01, Figure 1H), and there was a reduced level in the [C6] and [C7] groups but this was without statistical significance. In contrast, the levels of ALT were increased in all groups of healthy rats receiving PBR treatment compared with those in the [K] group and, especially, it was significantly increased in the [Z4] and [Z5] groups compared with animals in the [K] group (*P* < 0.05, Figure 1H). The TBIL level was reduced in all dose groups but without any significant difference compared with animals in the [CM] group (Figure 1I), except for the [C1] and [C6] groups. In contrast, there was a significantly increased TBIL level in the [Z1] ~ [Z4] groups (almost 3-fold) and the [Z5] group compared with the [K] group (*P* < 0.05, Figure 1I), while there was no significant difference in the elevated levels in the [Z6] and [Z7] groups compared with the [K] group. The serum levels of ALP, TBA, CREA, and BUN were reduced in both CUMS and healthy rats after PBR treatment with a low dose and increased with a high dose compared with rats not given PBR treatment (Figures 1J–M). However, the level of ALP in [C7] was higher compared with that in healthy rats given the same dose of PBR (Figure 1J). The level of BUN in the [Z7] group was significantly increased, almost 2-fold, and the TBA level in the [Z4] and [Z5] groups also showed a significant increase (*P* < 0.05, Figures 1M,K) compared with that in CUMS rats given the same dose of PBR. The AST level was significantly reduced in all groups of CUMS rats after PBR treatment (*P* < 0.05, Figure 1N). These results suggested that PBR in the concentration of D4 ~ D7 could cause liver injury in healthy rats, but did have not much effect in CUMS-induced rats.

### Effect on metabolites of rat serum by using the UHPLC- Q exactive orbitrap -MS method

#### Assessment of the stability of the UHPLC- Q exactive orbitrap -MS method

According to the results above, serum samples of rats in [CM], [C4], [C6], [C7], [K], [Z4], [Z6], and [Z7] groups were analyzed in both positive and negative modes by an UHPLC-Q Exactive Orbitrap-MS system for further metabolomic analysis of different physiological states. Typical total ion current (TIC) chromatograms of those are showed in Figure S1 (about 3,252 ions).

The LC–MS system stability for the large-scale sample analysis was demonstrated by a test of pooled QC samples. All the QC samples were observed tightly clustered in the result of principal components analysis (PCA). Moreover, 10 ions with different retention times and m/z values were extracted from the Base Peak Intensity chromatograph of each sample and selected for method validation. The retention time and m/z of 10 selected ions in eight QC samples also showed good system stability. The RSDs of the 10 ions were 0.04–0.67% for retention times, 1.40 × 10^−05^-1.52 × 10^−04^% for the m/z value (Table S1). These results indicated that the large-scale sample analysis had hardly any effect on the reliability of data.

#### Differential metabolic profile in CUMS-induced rats, with or without PBR

**Figure 2:**
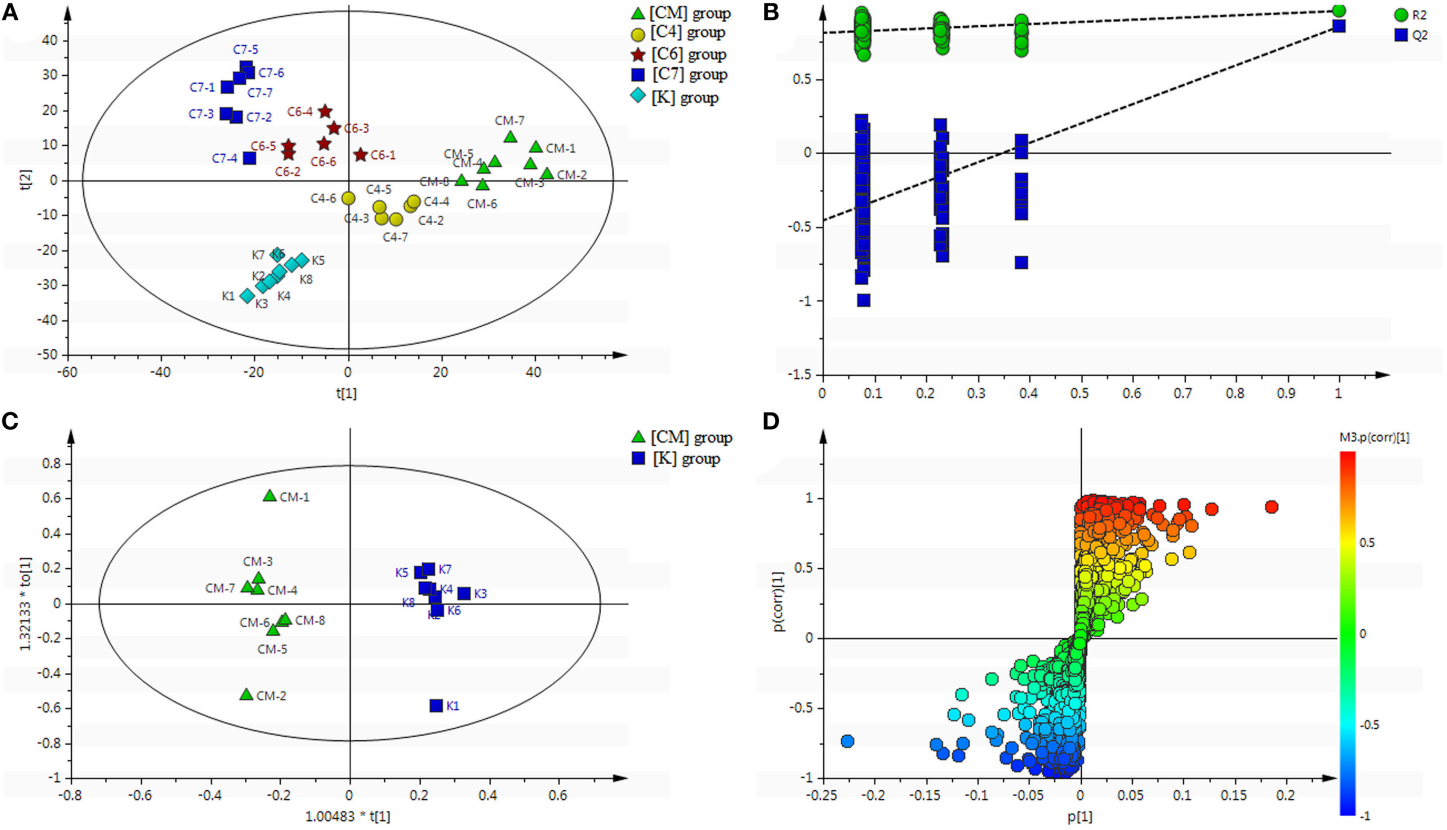
**(A)** Results of multiple pattern recognition of serum metabolites in CUMS rats with or without exposure to PBR. PLS-DA score plot (*n* = 8, R^2^Y = 0.981, R^2^X = 0.531, *Q*^2^ = 0.811). **(B)** Results of permutation tests. *R*^2^ = 0.802, *Q*^2^ = −0.498. **(C)** OPLS score plot (*n* = 8, R^2^Y = 0.977, R^2^X = 0.391, *Q*^2^ = 0.873) of [K] group and [CM] group. **(D)** OPLS S-plot of [K] group and [CM] group. Each triangle in the S-plot represents an ion. Ions far away from the origin were potential biomarkers.

All the discovery data set from the two ion scan modes were subjected to PLS-DA pattern recognition analysis after UV scaling to give a snapshot of the systematic metabolism in the [CM], [K], [C4], [C6], and [C7] groups. PLS-DA score plots showed an obvious separation between the [CM] and [K] (effects of CUMS-induced stress), [C4] and [CM], [C6] and [CM], as well as [C7] and [CM] (effects of PBR on CUMS-induced rats) groups (Figure 2A). The model parameters of R^2^Y and Q^2^ represent the reliability of the multiple pattern recognition methods. R^2^Y represents how well the interpretation of the data fits the model, and Q^2^ indicates the predictive accuracy of the model. The value of R^2^Y = 0.981 and *Q*^2^ = 0.811 demonstrated that the PLS-DA model could accurately describe the data. Furthermore, the results of the permutation tests showed that the two models were not overfitting but reflected the metabolic changes produced (intercepts: *R*^2^ = 0.802, *Q*^2^ = −0.498) (Figure 2B).

To fully differentiate between the metabolites in the [CM] and [K] groups, the OPLS-DA was used to eliminate unrelated variations contained within spectra. The OPLS-DA score plot shows a statistically significant difference in data between the [CM] and [K] groups (Figure 2C), suggesting that metabolic profiles significantly changed in rat serum with CUMS stress. The corresponding OPLS S-plot (Figure 2D) in turn shows the contribution of different variables and ions far away from the origin might be potential biomarkers.

**Figure 3:**
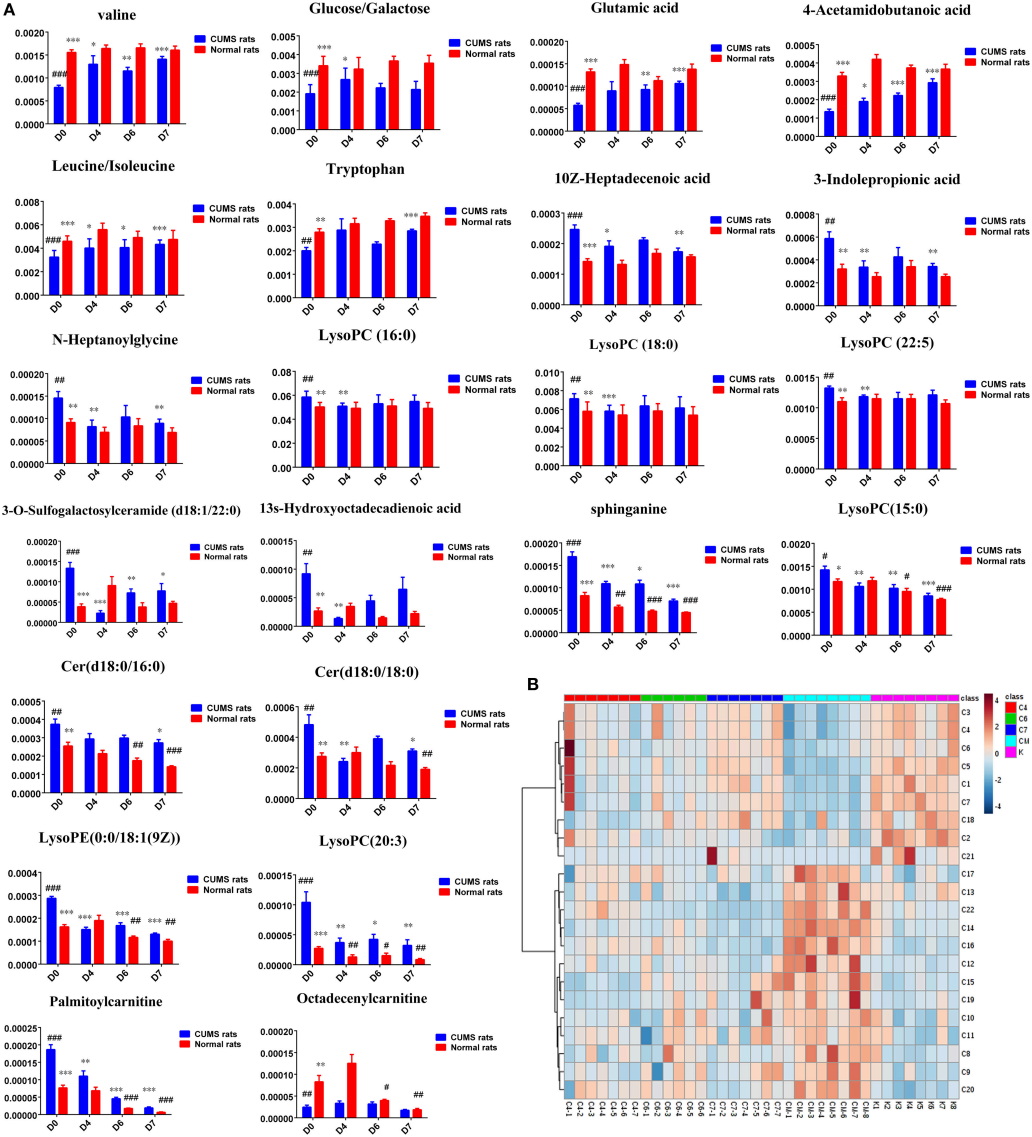
**(A)** Normalized intensity levels of differential metabolites in serum samples of CUMS rats (◼blue), and the relative content of the corresponding metabolites in normal rats (◼red). D0: administration of an equal volume of vehicle. D4: the concentration of PBR was 1.25 g/ml (expressed as the weight of raw materials). D6: the concentration of PBR was 5.0 g/ml. D7: the concentration of PBR was 10.0 g/ml. Data are presented as mean ± SEM. *n* = 8 each group. ^*^P < 0.05, ^**^P < 0.01, ^***^P < 0.001 vs. [CM] group; ^#^*P* < 0.05, ^##^*P* < 0.01, ^###^*P* < 0.001 vs. [K] group; **(B)** the relative content of the heatmap of differential metabolites in serum samples of CUMS rats with PBR treatment. Class C4: the [C4] group. Class C6: the [C6] group. Class C7: the [C7] group. Class CM: the [CM] group. Class K: the [K] group. The ribbon−4~4: represents the relative content of the differential metabolites from low to high.

**Figure 4:**
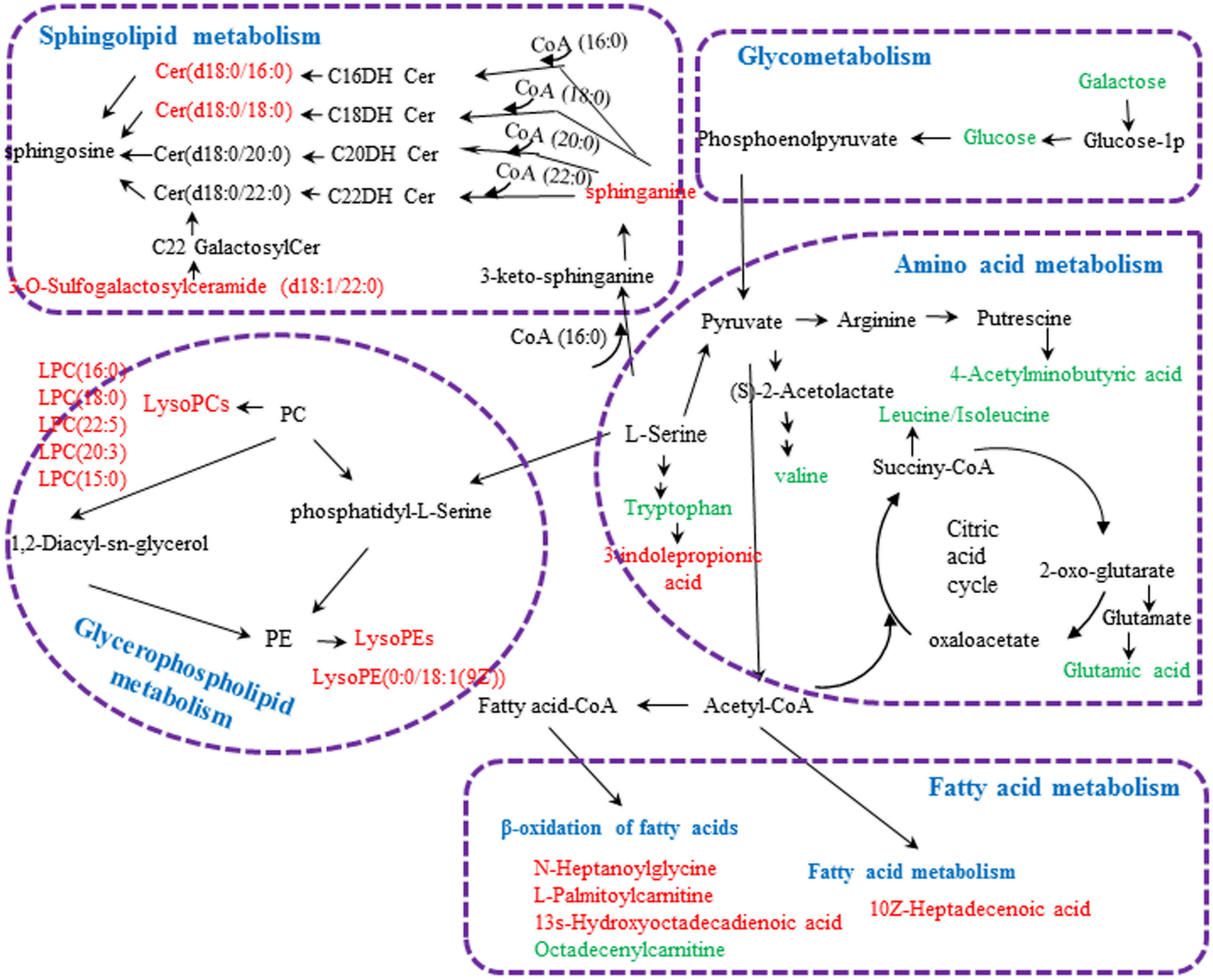
Metabolic pathways involved in the [CM] group vs. the [K] group. Red fonts represent up-regulated metabolites in the [CM] group vs. the [K] group; Green fonts represent down-regulated metabolites in the [CM] group vs. the [K] group. Blue fonts represent the metabolic pathway.

**Figure 5:**
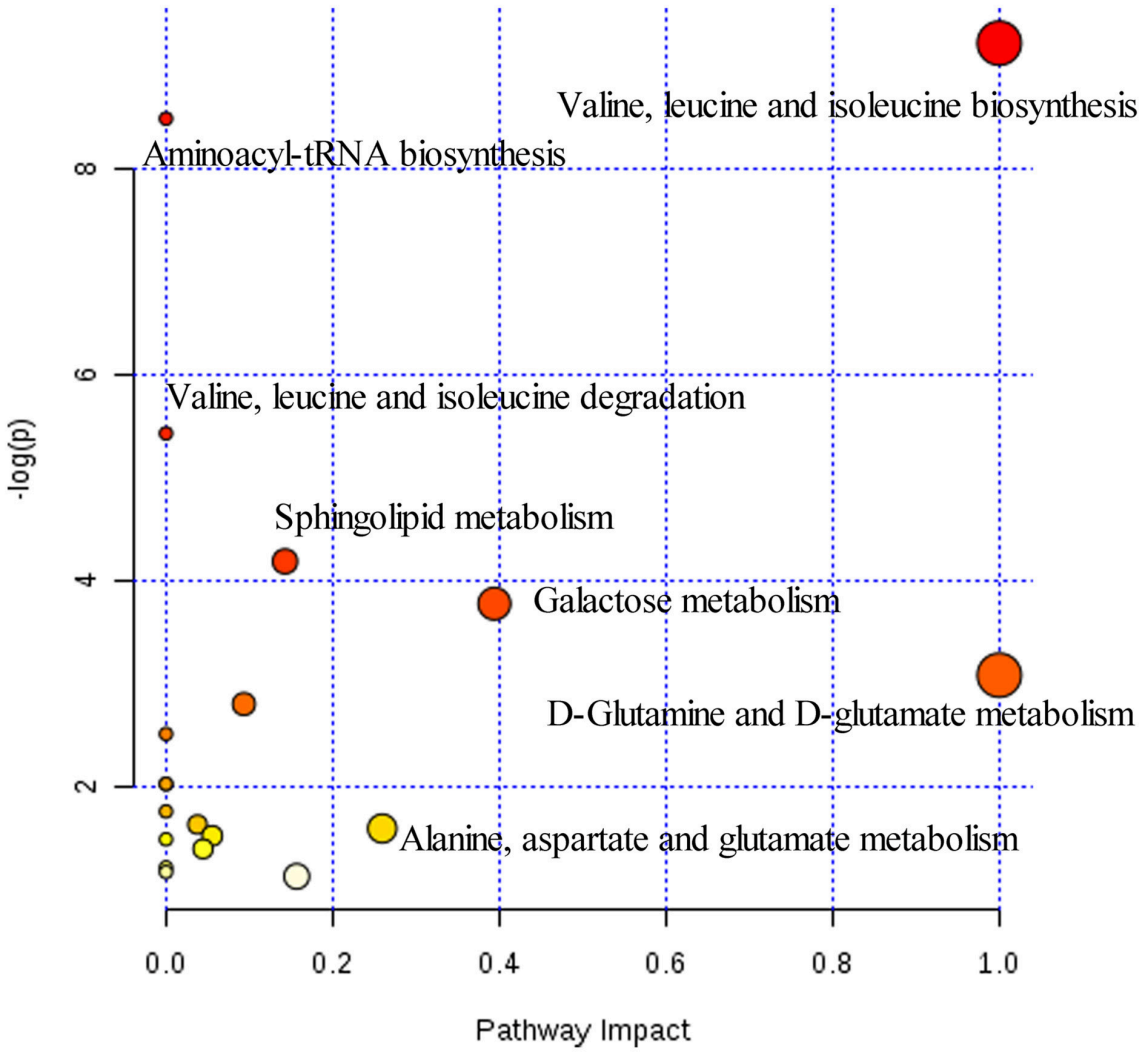
Summary of pathways analysis of CUMS rats with PBR treatment with MetaboAnalyst. Each point represents one metabolic pathway; the size of dot and shades of color are positively correlated with the impact on the metabolic pathway.

Twenty-three significantly differential metabolites were identified between the [CM] and [K] groups. The change in these specific metabolites in [CM] vs. [K] and in the drug-treatment groups compared with [CM] are clearly shown in Table 1 and the detailed change in the relative content of these specific metabolites is shown in Figure 3A. 4-Acetamidobutanoic acid, galactose/glucose, leucine, isoleucine, valine, tryptophan, octadecenylcarnitine and glutamic acid were significantly (0.38–0.68-fold) lower in the [CM] group compared with the [K] group (Figure 3A). In contrast, lyso-phosphatidylcholines (LPCs), sphingolipids, lyso-phosphatidylethanolamines (LPEs), fatty acids, palmitoylcarnitine and 3-indolepropionic acid were significantly (1.27–3.3-fold) higher in the [CM] group compared with the [K] group (Figure 3A). Also, all of these differential metabolites were regulated to a level close to the [K] group after PBR treatment in the [C4], [C6], and [C7] groups according to the relative content heatmaps of these differential metabolites (Figure 3B), except octadecenylcarnitine without reversed regulation at any dose of PBR and palmitoylcarnitine with an over-reversed regulation at the D7 dose. To further understand the correlation between these potential biomarkers, the established metabolic pathway was examined on the database sources of KEGG and the related literature reports. The markedly changed metabolites were associated with glycometabolism, amino acid metabolism, glycerophospholipid metabolism, sphingolipid metabolism and fatty acid metabolism (Figure 4). Based on the analysis with MetaboAnalyst (Figure 5), seven of the metabolic pathways with an impact value >0.1 and –logP >4 were considered the most pertinent in CUMS rats with PBR treatment, including valine, leucine and isoleucine biosynthesis, valine, leucine and isoleucine degradation, D-Glutamine and D-Glutamine metabolism, Alanine, aspartate and glutamate metabolism, Aminoacyl-TRNA biosynthesis, Galactose metabolism and Sphingolipid metabolism, which are involved in amino acid metabolism, glycometabolism and sphingolipid metabolism.

**Table 1:** Identified differential metabolites in the serum of CUMS rats with or without administration of PBR.

**No**.	**Metabolites**	**T_R_ (min)**	**m/z**	**ion**	**CM/K**	**C7/CM**	**C6/CM**	**C4/CM**	**Metabolic pathway**
C1	4-Acetamidobutanoic acid	1.18	146.0812	M+H	↓^a^	↑^a^	↑^a^	↑^a^	Arginine and proline metabolism
C2	Glucose/Galactose^b^	1.34	179.0553	M-H	↓^a^	↑	↑	↑^a^	Glycometabolism
C3	Leucine^b^	3.01	132.1025	M+H	↓^a^	↑^a^	↑^a^	↑^a^	Amino acid metabolism
C4	Isoleucine ^b^	3.22	132.1025	M+H	↓^a^	↑^a^	↑^a^	↑^a^	Amino acid metabolism
C5	Valine^b^	1.67	118.0864	M+H	↓^a^	↑^a^	↑^a^	↑^a^	Amino acid metabolism
C6	Tryptophan^b^	5.99	205.0976	M+H	↓^a^	↑^a^	↑	↑	Tryptophan metabolism
C7	Glutamic acid^b^	1.38	146.0453	M-H	↓^a^	↑^a^	↑^a^	↑	Amino acid metabolism
C8	3-Indolepropionic acid	9.71	190.0868	M+H	↑^a^	↓^a^	↓	↓^a^	Tryptophan metabolism
C9	LysoPC (16:0)	25.13	496.3402	M+H	↑^a^	↓	↓	↓^a^	Glycerophospholipid metabolism
C10	LysoPC (18:0)	27.65	524.3715	M+H	↑^a^	↓	↓	↓^a^	Glycerophospholipid metabolism
C11	LysoPC (22:5)	28.30	568.3618	M-H	↑^a^		↓	↓^a^	Glycerophospholipid metabolism
C12	LysoPC(20:3)	25.24	546.3531	M+H	↑^a^	↓^a^	↓^a^	↓^a^	Glycerophospholipid metabolism
C13	LysoPC(15:0)	28.36	482.3245	M+H	↑^a^	↓^a^	↓^a^	↓^a^	Glycerophospholipid metabolism
C14	LysoPE[0:0/18:1(9Z)]	26.24	480.3452	M+H	↑^a^	↓^a^	↓^a^	↓^a^	Glycerophospholipid metabolism
C15	3-O-Sulfogalactosylceramide (d18:1/22:0)	23.82	862.5595	M-H	↑^a^	↓^a^	↓^a^	↓^a^	sphingolipid metabolism
C16	Sphinganine	18.02	302.3058	M+H	↑^a^	↓^a^	↓^a^	↓^a^	sphingolipid metabolism
C17	Cer(d18:0/16:0)	30.08	540.5354	M+H	↑^a^	↓^a^	↓	↓	sphingolipid metabolism
C18	Cer(d18:0/18:0)	30.90	568.5667	M+H	↑^a^	↓^a^	↓	↓	sphingolipid metabolism
C19	13s-Hydroxyoctadecadienoic acid	22.65	297.2431	M+H	↑^a^	↓^a^		↓^a^	Fatty acid metabolism
C20	10Z-Heptadecenoic acid	28.57	267.2112	M-H	↑^a^	↓^a^		↓^a^	Fatty acid metabolism
C21	N-Heptanoylglycine	9.11	186.0553	M-H	↑^a^	↓^a^			β-oxidation of fatty acid
C22	Palmitoylcarnitine	22.08	400.3426	M+H	↑^a^	↓^a^	↓^a^	↓^a^	β-oxidation of fatty acid
C23	Octadecenylcarnitine	22.72	426.358	M+H	↓^a^				β-oxidation of fatty acid

*↑shows up-regulated metabolite; ↓shows down-regulated metabolite*.

a *Means a statistically significant difference at p < 0.05*.

b *Validated with standard. [CM] group compared with [K] group, CM/K; [C4] group compared with [CM] group, C4/CM; [C6] group compared with [CM] group, C6/CM; [C7] group compared with [CM] group, C7/CM*.

#### Differential metabolic profile in healthy control rats, with or without PBR

**Figure 6:**
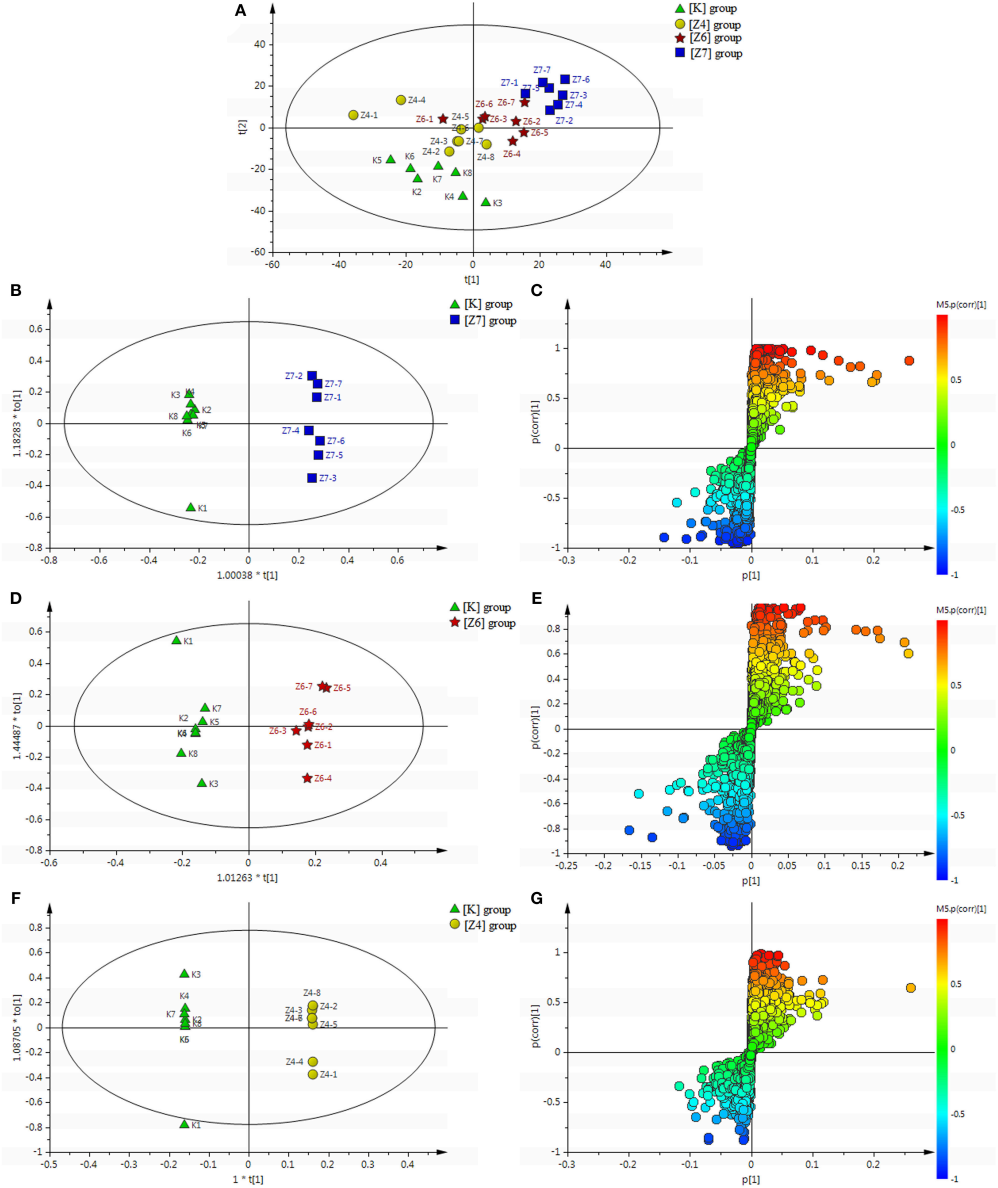
**(A)** Results of multiple pattern recognition of serum metabolites of healthy rats with or without exposure to PBR. PCA score plot (*n* = 8, R^2^X = 0.453, *Q*^2^ = 0.0152). **(B)** OPLS score plot (*n* = 8, R^2^Y = 0.997, R^2^X = 0.0.504, *Q*^2^ = 0.873) of [Z7] group and [K] group and the corresponding OPLS S-plot **(C)**. **(D)** OPLS score plot (*n* = 8, R^2^Y = 0.974, R^2^X = 0.0.29, *Q*^2^ = 0.558) of [Z6] group and [K] group and the corresponding OPLS S-plot **(E)**. **(F)** OPLS score plot (*n* = 8, R^2^Y = 1, R^2^X = 0.699, *Q*^2^ = 0.505) of [Z4] group and [K] group and the corresponding OPLS S-plot **(G)**. Each triangle in the S-plot represents an ion. Ions far away from the origin were potential biomarkers.

PCA scores plots showed that there was a significant separation among the [K], [Z4], [Z6], and [Z7] groups and it was most significant between the [Z7] and [K] groups (Figure 6A), suggesting that PBR had an influence on the metabolic profile of normal rats. R^2^Y = 0.997 and *Q*^2^ = 0.873 in the OPLS models in [Z7] vs. [K] demonstrated that the models were reliable (Figure 6B). The corresponding OPLS S-plots showed the contribution of different variables in [Z7] vs. [K] (Figure 6C). The analysis in [Z6] and [Z4] vs. [K] showed the similar results (Figures 6D–G).

**Figure 7:**
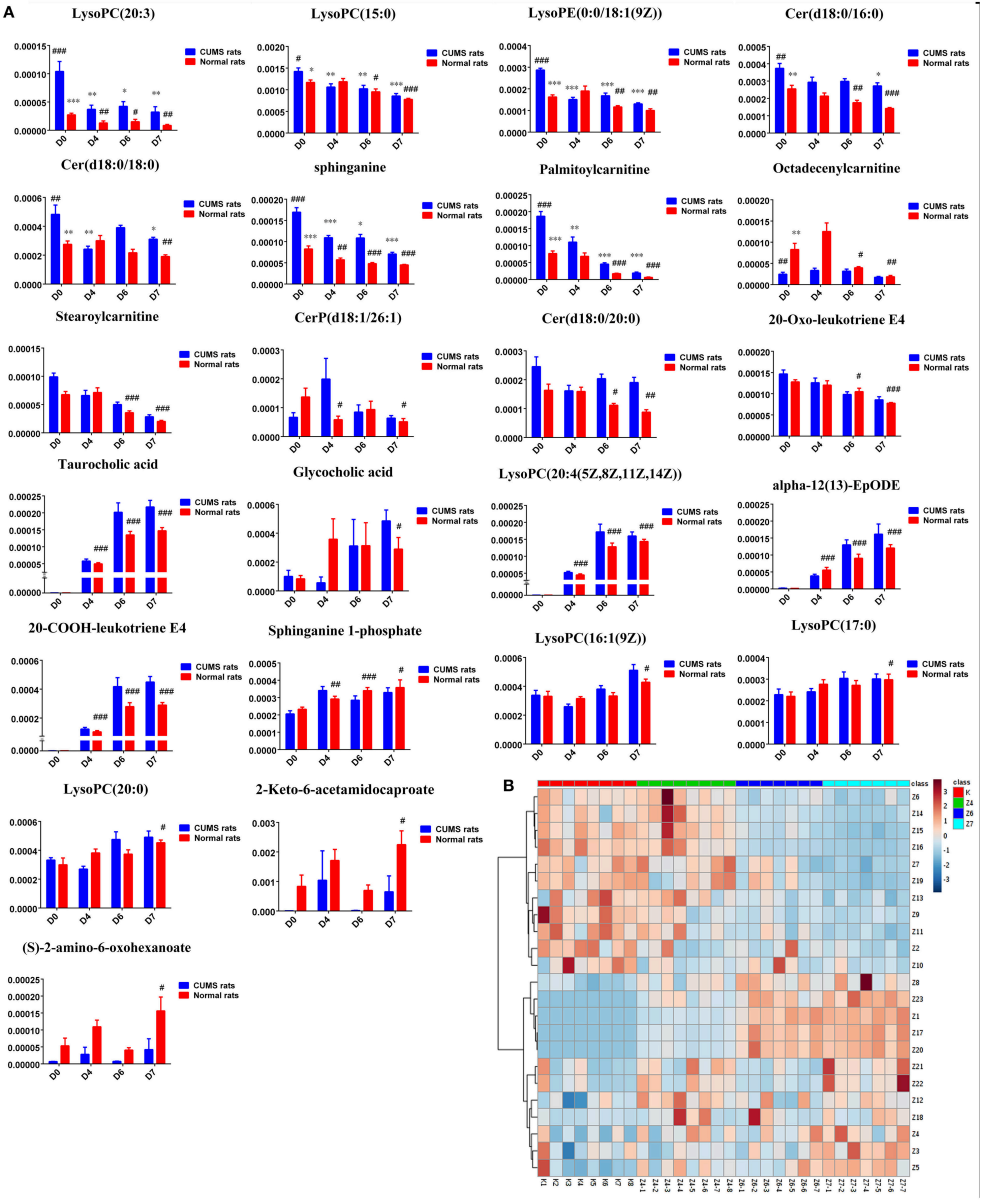
**(A)** Normalized intensity levels of potential biomarkers in serum samples of normal rats (◼red), and the relative content of the corresponding metabolites in CUMS rats (◼blue). D0: administration of equal volumes of vehicle. D4: the concentration of PBR was 1.25 g/ml (expressed as the weight of raw materials). D6: the concentration of PBR was 5.0 g/ml. D7: the concentration of PBR was 10.0 g/ml. Data are presented as mean ± SEM. *n* = 8 each group. ^*^P < 0.05, ^**^P < 0.01, ^***^P < 0.001 vs. [CM] group; ^#^*P* < 0.05, ^##^*P* < 0.01, ^###^*P* < 0.001 vs. [K] group. **(B)**: the relative content of the heatmap of differential metabolites in serum samples of healthy rats with PBR administration. Class K: the [K] group. Class Z4: the [Z4] group. Class Z6: the [Z6] group. Class Z7: the [Z7] group. The ribbon −3~3: represents the relative content of the differential metabolites from low to high.

**Figure 8:**
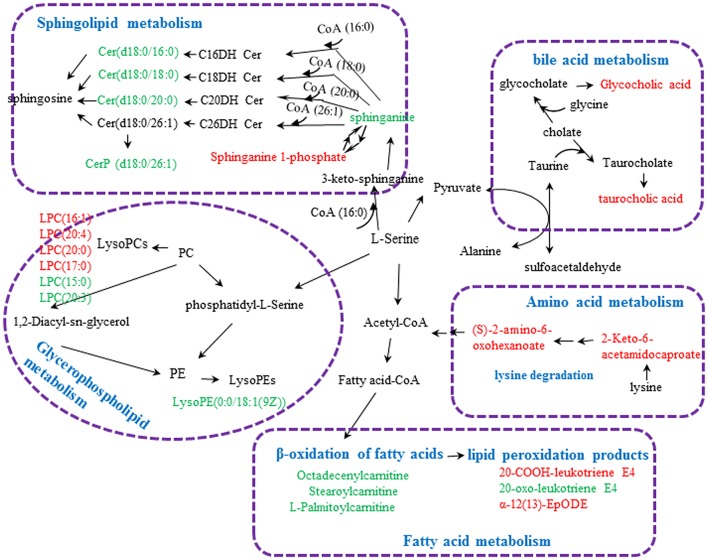
Metabolic pathways involved in the [Z4], [Z6], or [Z7] group vs. the [K] group. Red fonts represent up-regulated metabolites in the [Z4], [Z6], or [Z7] group vs. the [K] group; Green fonts represent down-regulated metabolites in the [Z4], [Z6], or [Z7] group vs. the [K] group. Blue fonts represent the metabolic pathway.

**Figure 9:**
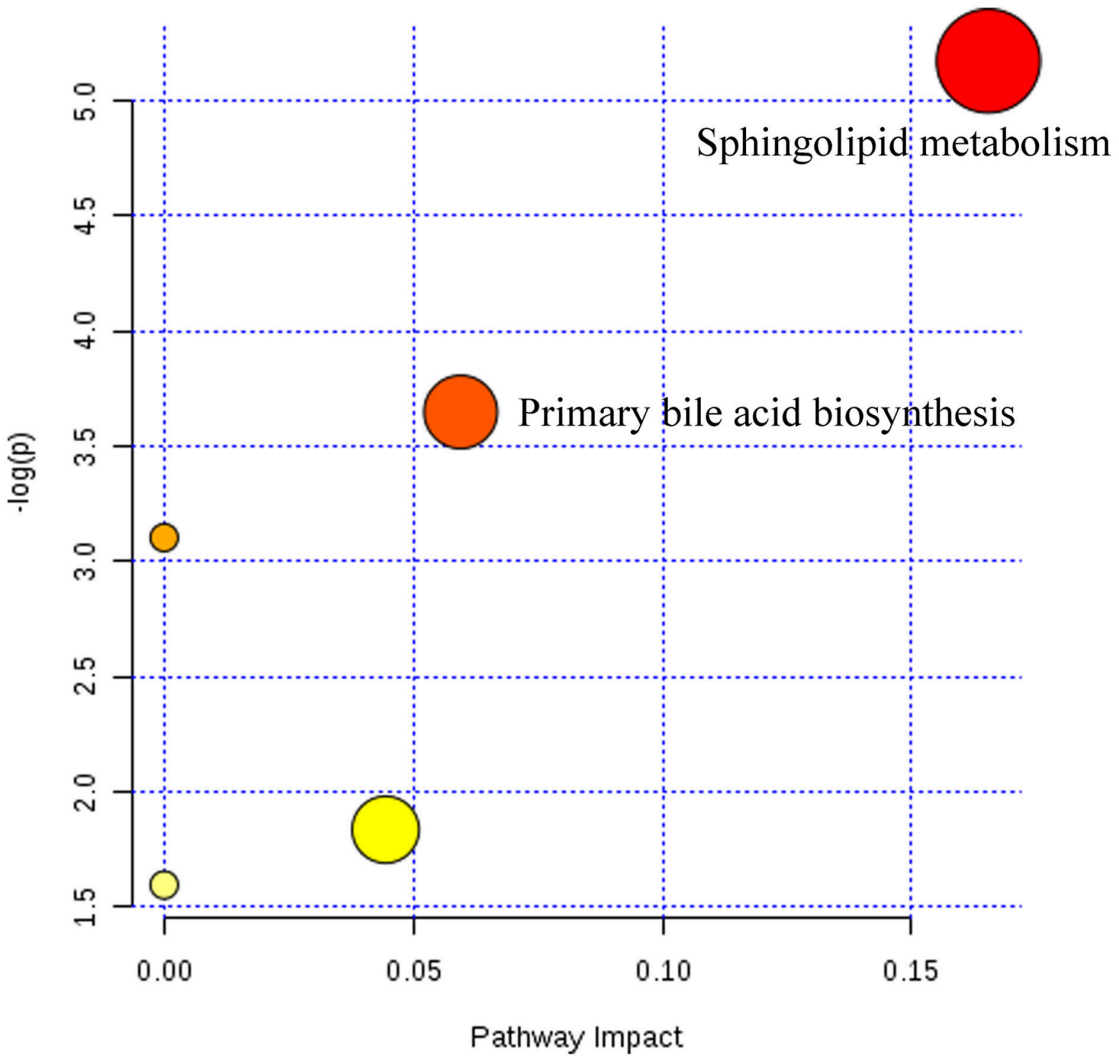
Summary of pathways analysis of healthy rats with PBR administration with MetaboAnalyst. Each point represents one metabolic pathway; the size of dot and shades of color are positively correlated with the impact of the metabolic pathway.

Twenty-three metabolites were modulated by PBR in healthy rats. The detailed information on these metabolites is shown in Table 2 and the detail change in relative content and the relative content of heatmaps of these specific metabolites are shown in Figure 7. Acyl carnitines, LPC (15:0), LPC (20:5), LysoPE[0:0/18:1(9Z)], 20-oxo-leukotriene E4 and sphingolipids, except for sphinganine 1-phosphate, were significantly (0.18–0.72-fold) lower in the [Z4], [Z6], or [Z7] groups compared with the [K] group (Figure 7). In contrast, the other metabolites, which included LPC (16:1), LPC (20:4), LPC (20:0), LPC (17:0), sphinganine 1-phosphate, taurocholic acid, Glycocholic acid, (S)-2-amino-6-oxohexanoate, 2-Keto-6-acetamidocaproate, 20-COOH-leukotriene E4, and α-12(13)-EpODE, were significantly (1.46–14-fold) higher in the [Z4], [Z6], or [Z7] groups compared with the [K] group (Figure 7). The markedly changed metabolites were associated with lysine degradation,glycerophospholipid metabolism, sphingolipid metabolism, fatty acid metabolism and bile acid metabolism (Figure 8). Based on the analysis using MetaboAnalyst (Figure 9), two of the metabolic pathways with an impact value >0.05 were considered as the most pertinent in healthy rats given PBR, including sphingolipid metabolism and primary bile acid biosynthesis.

**Table 2:** Identified differential metabolites in the serum of healthy rats with or without administration of PBR.

**No**.	**Metabolites**	**T_R_ (min)**	**m/z**	**ion**	**Z4/K**	**Z6/K**	**Z7/K**	**Metabolic pathway**
Z1	LysoPC(20:4)	18.92	544.2673	M+H	↑^a^	↑^a^	↑^a^	Glycerophospholipid metabolism
Z2	LysoPC(20:3)	25.24	546.3531	M+H	↓	↓	↓^a^	Glycerophospholipid metabolism
Z3	LysoPC[16:1(9Z)]	22.66	494.3248	M+H			↑^a^	Glycerophospholipid metabolism
Z4	LysoPC(17:0)	26.78	510.3564	M+H	↑	↑	↑^a^	Glycerophospholipid metabolism
Z5	LysoPC(20:0)	30.79	552.4027	M+H	↑	↑	↑^a^	Glycerophospholipid metabolism
Z6	LysoPE[0:0/18:1(9Z)]	26.24	480.3452	M+H		↓^a^	↓^a^	Glycerophospholipid metabolism
Z7	LysoPC(15:0)	27.73	482.3245	M+H		↓^a^	↓^a^	Glycerophospholipid metabolism
Z8	Sphinganine 1-phosphate	22.44	382.2721	M+H	↑^a^	↑^a^	↑^a^	sphingolipid metabolism
Z9	sphinganine	18.02	302.3058	M+H		↓^a^		sphingolipid metabolism
Z10	CerP(d18:1/26:1)	30.70	784.5778	M+H	↓^a^		↓^a^	sphingolipid metabolism
Z11	Cer(d18:0/16:0)	30.08	540.5354	M+H		↓^a^	↓^a^	sphingolipid metabolism
Z12	Cer(d18:0/18:0)	30.90	568.5667	M+H		↓	↓^a^	sphingolipid metabolism
Z13	Cer(d18:0/20:0)	31.75	596.5979	M+H		↓^a^	↓^a^	sphingolipid metabolism
Z14	Octadecenylcarnitine	22.72	426.358	M+H	↓	↓^a^	↓^a^	β-oxidation of fatty acid
Z15	Stearoylcarnitine	24.55	428.374	M+H		↓^a^	↓^a^	β-oxidation of fatty acid
Z16	Palmitoylcarnitine	22.08	400.3426	M+H		↓^a^	↓^a^	β-oxidation of fatty acid
Z17	Taurocholic acid ^b^	16.80	514.221	M-H	↑^a^	↑^a^	↑^a^	bile acid metabolism
Z18	Glycocholic acid ^b^	14.97	464.3014	M-H	↑	↑	↑^a^	bile acid metabolism
Z19	20-Oxo-leukotriene E4	24.45	454.2932	M+H			↓^a^	leukotriene metabolism
Z20	20-COOH-leukotriene E4	16.79	470.2309	M+H	↑^a^	↑^a^	↑^a^	leukotriene metabolism
Z21	2-Keto-6-acetamidocaproate	6.01	188.0706	M+H	↑		↑^a^	Lysine metabolism
Z22	(S)-2-amino-6-oxohexanoate	5.52	144.045	M-H	↑		↑^a^	Lysine metabolism
Z23	α-12(13)-EpODE	16.01	293.1756	M-H	↑^a^	↑^a^	↑^a^	lipid metabolism

*↑ shows up-regulated metabolite; ↓ shows down-regulated metabolite*.

a *Means a statistically significant difference at p < 0.05*.

b *Validated with standard. [Z4] group compared with [K] group, Z4/K; [Z6] group compared with [K] group, Z6/K; [Z7] group compared with [K] group, Z7/K*.

#### Metabolic network analysis in both CUMS and healthy rats with or without PBR

**Figure 10:**
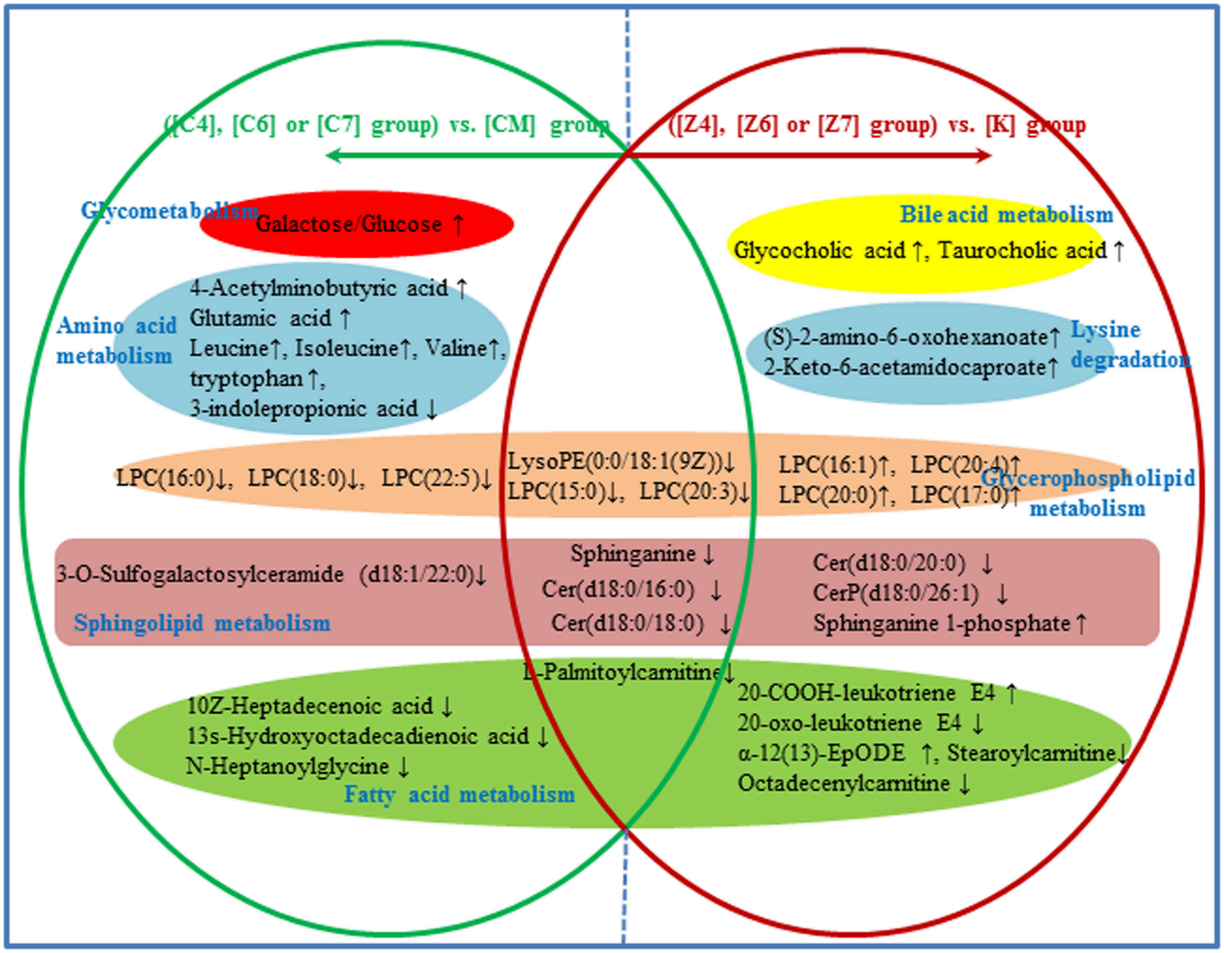
Different metabolites and corresponding pathways in CUMS rats or healthy rats following PBR administration. “↑” and “↓” represent that the metabolite is up- or down-regulated in CUMS rats or healthy rats folloowing PBR administration. In CUMS rats following PBR administration ([C4], [C6], and [C7]), glycometabolism and amino acid metabolism were up-regulated; however, glycerophospholipid metabolism, sphingolipid metabolism and fatty acid metabolism were down-regulated, all being compared with the corresponding group without drug treatment [CM]. In healthy rats following PBR administration ([Z4], [Z6], and [Z7]), lysine degradation and bile acid metabolism were up-regulated; however, the other three pathways of glycerophospholipid metabolism, sphingolipid metabolism and fatty acid metabolism were up- or down-regulated, all being compared with the corresponding group without drug treatment [K].

In the 38 differential metabolites in both CUMS rats and healthy rats given PBR, 15 of the metabolites reversed to normal after PBR treatment of CUMS-induced depression, while they were not significantly affected in healthy rats given PBR (Figure 10). This indicated that these 15 metabolites, including galactose/glucose, 4-acetamidobutanoic acid, glutamic acid, leucine, isoleucine, valine, LPC (16:0), LPC (18:0), LPC (22:5), N-heptanoylglycine, tryptophan, 3-indolepropionic acid, 10Z-heptadecenoic acid, 3-O-Sulfogalactosylceramide and 13s-hydroxyoctadecadienoic acid, might be potential biomarkers most highly correlated with the pharmacological effects of PBR. The 15 metabolites were associated with glycometabolism,amino acid metabolism,glycerophospholipid metabolism and fatty acid metabolism (Figure 10).

Another 7 metabolites which included sphinganine, Cer(d18:0/16:0), Cer(d18:0/18:0), LPC(15:0), LPC (20:3), LPE(0:0/18:1(9Z), and palmitoylcarnitine, were the same regulative differential metabolites and showed the same down-regulation in both CUMS rats and healthy rats given PBR treatment (*P* < 0.05, Figure 10). One of these metabolites (Octadecenylcarnitine) was significantly reduced in the [CM] group compared with the [K] group (*P* < 0.05, Figure 10), but there was no reversed regulation in CUMS rats after PBR treatment. There was also a significant reduction in groups of healthy rats given PBR compared with the [K] group. These 8 metabolites were associated with glycerophospholipid metabolism, sphingolipid metabolism and fatty acid metabolism (Figure 10).

Another 15 of these metabolites were only significantly altered in healthy rats after PBR treatment (Figure 10), including LysoPC(20:4), LysoPC[16:1(9Z)], LysoPC(17:0), LysoPC(20:0), Sphinganine 1-phosphate, Cer(d18:0/20:0), Stearoylcarnitine, Taurocholic acid, Glycocholic acid, 20-Oxo-leukotriene E4, 20-COOH-leukotriene E4, 2-Keto-6-acetamidocaproate, (S)-2-amino-6-oxohexanoate, and α-12(13)-EpODE. These 15 metabolites were associated with lysine degradation, glycerophospholipid metabolism, sphingolipid metabolism, fatty acid metabolism and bile acid metabolism (Figure 10).

**Figure 11:**
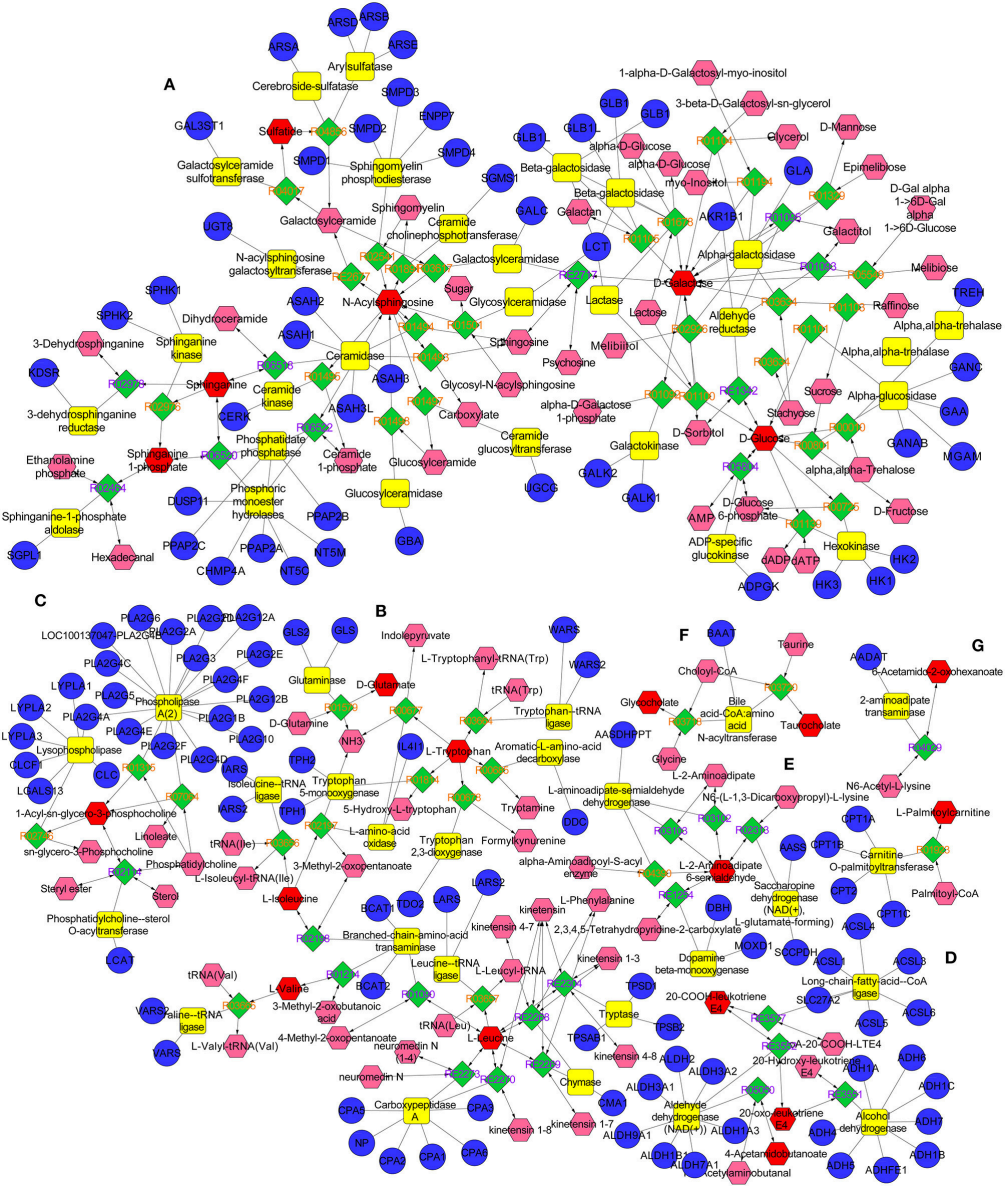
The metabolic networks involved in enzymes and genes were established based on the differential metabolites in both CUMS and healthy rats following PBR treatment. The metabolic networks of sphingolipid metabolism **(A)**, glycometabolism **(A)**, amino acid metabolism **(B,E)**, glycerophospholipid metabolism **(C)**, fatty acid metabolism **(D,G)** and bile acid metabolism **(F)**.

The metabolic networks involved in some enzymes and genes were constructed with the use of Cytoscape to better understand the internal correlation of the potential biomarkers in terms of enzyme or gene levels. The metabolic networks which were established based on the markedly differential metabolites are shown in Figure 11. The sphingomyelinases, ceramide kinase, sphinganine kinase, and others involved in in sphingolipid metabolism are shown (Figure 11A). Some enzymes and genes were also found to be found involved in amino acid metabolism (Figure 11B) and the phospholipase A2 (PLA2) which is encoded by the PLA2G family of genes is involved in glycerophospholipid metabolism (Figure 11C). The long-chain-fatty-acid-CoA ligase, aldehyde dehydrogenase [NAD (+)] and alcohol dehydrogenase are involved in leukotriene production (Figure 11D). Bile acid-CoA: amino acid N-acyltransferase encoded by gene of BAAT was also found to be involved in bile acid metabolism (Figure 11F). Carnitine palmitoyltransferase II was also found to be involved in the production of palmitoylcarnitine which was involved in the β-oxidation of fatty acids (Figure 11G).

## Discussion

We are the first group to report that PBR administration produces differential effective and toxic reactions in CUMS (with depression) and healthy rats. PBR treatment significantly reversed depression in the CUMS-induced rats in this study. The altered lysine degradation, sphingolipid metabolism, glycerophospholipid metabolism, fatty acid metabolism, and bile acid metabolism could be at least partly responsible for the systemic toxicity in healthy rats produced by PBR. This in fact confirms the use of PBR only in subjects with a particular physical condition.

Bupleuri Radix is recorded as the highest grade of herbal drug in Shennong's Materia Medica and exhibits liver relief and hepatoprotective effects. It is frequently contained in Chinese herbal formulas as a major component of the prescription for the treatment of depression especially (Zhou et al., 2011; Su et al., 2014). However, it has also been recorded that Bupleuri Radix can cause acute liver injury, hepatocyte apoptosis and acute hepatitis if give in an overdose or on long-term treatment (Hsu et al., 2006; Yang et al., 2017). In the present study, the differential effective and toxic responses of PBR, produced by the main drug of the XYS formula, was investigated in CUMS and healthy rats. The CUMS-induced rats effectively mimic depression in humans as reflected in a loss in body weight, reduced sucrose intake in the sucrose preference test and reduced locomotor activity in the open-field test (Willner, 1997). Our biochemical findings indicate that PBR can produce hepatic or renal damage in healthy control rats but it has a comparatively mild adverse effect in CUMS rats (with a depression pattern). To further examine the detailed mechanisms of the differential effects and toxic responses to PBR in CUMS and healthy rats, a LC-MS metabolomics approach was employed to determine the global serum metabolic profiles, and the metabolic pathways and networks involved were analyzed.

The improved results of the behavior research suggest that PBR has a positive antidepressant effect in depressed subjects. Most of the altered metabolites in CUMS rats became reversed after PBR treatment. Our findings indicate that energy production is perturbed, such as abnormal glycometabolism, by down-regulating glucose/galactose. Glucose/galactose can be metabolized by glycolysis to produce pyruvate, ATP and lactate, which act as energy substrates (Haas et al., 2016). Pyruvate, produced during the process of glycolysis, can then be converted to acetyl coenzyme A, which ultimately feeds into the TCA cycle (Haas et al., 2016). The down-regulated plasma glucose/galactose suggest that glycolysis is disturbed, resulting in an insufficient supply of energy substrate for the energy production pathway, leading to further energy deficiency. The down-regulated glycometabolism was consistent with a previous study on depression (Liu C. C. et al., 2015), and energy deficiency associated with depression has been reported in previous studies (Hundal, 2007; Liu C. C. et al., 2015). In addition to glycometabolism by down-regulating glucose/galactose, CUMS rats were also characterized by an alteration in amino acid metabolism, with down-regulated 4-acetamidobutanoic acid, glutamic acid, leucine, isoleucine, valine, tryptophan as well as up-regulated 3-indolepropionic acid. Glutamatergic transmission mediated by astrocytes is implicated in the etiology of depression (Wang et al., 2017) and glutamate-glutamine cycling can provide glutamine, which is a precursor of gamma aminobutyric acid (GABA) synthesis (Wang et al., 2017). The reduced level of glutamic acid, an excitatory neurotransmitter, in plasma in CUMS rats was consistent with previous studies and it plays a role in depression (Ni et al., 2008; Yang et al., 2014). In addition, 4-acetamidobutanoic acid, an important intermediate in arginine and proline metabolism, is also a precursor of GABA. The reduced levels of 4-acetamidobutanoic acid and glutamic acid might lead to disruption of the GABAergic system which would potentially contribute to the etiology of major depressive disorder (Pehrson and Sanchez, 2015; Wang et al., 2017). Leucine, isoleucine and valine are branched-chain amino acids and it has been hypothesized that such branched-chain amino acids are transported via a blood–brain carrier system associated with 5-HT synthesis (Ni et al., 2008; Liu C. C. et al., 2015). The reduced concentration of branched-chain amino acids in plasma could be an indication of the disturbed release of brain 5-HT, which is closely related to central fatigue (Blomstrand, 2006), a common symptom of depression. In addition, isoleucine can maintain the physiological function of nitrogen balance in the brain, while an imbalance of nitrogen in the brain affects normal operation of the central nervous system (Shimomura and Harris, 2006). It can also increase the expression of hippocampal BDNF cells *in vitro*, and the BDNF central nervous system is an important neurotrophic factor in the signal pathways involved in depression (Furukawa-Hibi et al., 2011). 3-Indolepropionic acid is a deamination product of L-tryptophan formed by commensal bacteria in human and animal intestines which then diffuses into the blood (Morita et al., 1992; Chyan et al., 1999) and tryptophan is a precursor of serotonin biosynthesis and up-regulated 3-indolepropionic acid is involved in tryptophan metabolism. An increased rate of tryptophan degradation reduces tryptophan levels and is associated with the reduction in serotonergic biosynthesis and may subsequently affect the function of the serotonergic system, ultimately contributing to depression (Umehara et al., 2017). Alterations of amino acid levels in depression are consistent with a previous study, indicating that amino-acid neurotransmitter system dysfunction plays a role in the pathophysiology of depression (Ni et al., 2008). PBR caused up-regulation of down-regulated glucose/galactose and amino acids to a level close to normal in CUMS rats while, in healthy rats, these metabolites remained unaltered following PBR administration. These results imply that these metabolites are involved in glycometabolism and amino acid metabolism and may be potential biomarkers most highly correlated with the pharmacological effects of PBR.

Except for altered glycometabolism and amino acid metabolism, sphingolipid metabolism, glycerophospholipid metabolism and fatty acid metabolism were similarly perturbed pathways in both CUMS rats and healthy rats following PBR administration. However, the regulated metabolic targets of PBR were not all identical in the different physiological conditions. CUMS rats were also characterized by alteration of sphingolipid metabolism and glycerophospholipid metabolism with up-regulated sphingolipids and lyso-glycerophospholipids. Sphingolipids are a major lipid class that constitute a significant proportion of brain membrane lipids, including ceramides, sphingomyelins, cerebrosides, gangliosides, and sphingosines (Abe and Norton, 1974; Müller et al., 2015; Dinoff et al., 2017). Ceramides (N-acylsphingosine), the central molecule in sphingolipid metabolism, are generated by hydrolysis of the major membrane sphingolipid, sphingomyelin, by acid or neutral sphingomyelinases (aSMase), by degradation of complex glycosphingolipids and by *de novo* synthesis from palmitoyl-CoA and serine (Jernigan et al., 2015; Ordóñez et al., 2016). They can be degraded by the catabolic route to sphingosine by ceramidases, and further phosphorylation to sphingosine 1-phosphate (S1P) (Ordóñez et al., 2016). Sphinganine, which can also be phosphorylated to sphinganine 1-phosphate, is an intermediate in the *de novo* synthesis from palmitoyl-CoA and serine, and is further transformed into ceramides (Ordóñez et al., 2016). An elevated level of sphingolipids has been implicated in depression (Dinoff et al., 2017). Gracia-Garcia et al. reported that plasma concentrations of ceramides were found to be elevated in individuals with depression or individuals that had experienced a depressive episode within the last 2 years when compared with non-depressed individuals (Gracia-Garcia et al., 2011; Dinoff et al., 2017). One potential neurobiological mechanism is that elevated ceramides may potentially lead to reduced hippocampal neurogenesis, resulting in hypothalamic-pituitary-adrenal (HPA) axis hyperactivity (Dinoff et al., 2017), while hyperactivity of the HPA axis has been found in depression (Stetler and Miller, 2011). Another study showed ceramides may alter monoamine neurotransmitter transport and signaling, which subsequently leads to a reduction in serotonergic transmission (Riddle et al., 2003). Further evidence that depression is associated with ceramides is that mice overexpressing aSMase displayed greater ceramide production, and exhibited depression-like behavior in several behavioral tests (Gulbins et al., 2013). Conversely, aSMase knockout mice presented with reduced total ceramide concentrations and displayed reduced depression-related behavior when compared with wild type mice (Gulbins et al., 2013). Moreover, many antidepressants inhibit acid sphingomyelinases, such as tricyclic antidepressants, fluoxetine and sertraline, further suggesting a role of aSMase and ceramides in depression and depression treatment (Kornhuber et al., 2008, 2010). In addition, sphingolipids are major constituents of eukaryotic cell membranes (Ordóñez et al., 2016) and, in addition to a structural role, some sphingolipids are bioactive and control vital biological functions by regulating signal transduction pathways involved in several processes (i.e., apoptosis, adhesion, autophagy, cell proliferation, differentiation, migration, and senescence)(Ordóñez et al., 2016). Kolesnick has reported that aSMase is activated in response to tumor necrosis factor (TNF-α) and other cytokines (Kolesnick and Kronke, 1998). The activation of ASMase and generation of C16-ceramide contributes to TNF-α-induced hepatocyte apoptosis and this is evidence that the dynamic balance between the intracellular levels of ceramide and S1P (the “ceramide/S1P rheostat”) may determine cell survival (Osawa et al., 2005). Lyso-glycerophospholipids are metabolic intermediates during glycerophospholipid hydrolysis by phospholipases A2 (PLA2) and phospholipids are a class of lipids that are also a major component of all cell membranes as they can form lipid bilayers (Jia et al., 2016). Abnormal glycerophospholipid metabolism and increased activity of A2 phospholipases are involved in the pathophysiology of depression and bipolar disorders (Kato et al., 1993; Papadimitriou et al., 2003). Studies have reported that lysophosphatidylcholines (LysoPCs) and lysophosphatidylethanolamines (LysoPEs) are increased significantly in the plasma of depressed patients (Liu X. et al., 2015) and higher serum LPC levels can increase oxidative stress by an activated 5-lipoxygenase pathway(Zou et al., 2009), while activation of inflammatory and oxidative nitrosative stress (IO&NS) pathways may contribute to a risk for major depression and cardiovascular disorders due to increased levels of pro-inflammatory cytokines and reactive oxygen species and reduced antioxidant defense in both disorders (Maes et al., 2011a,b). Therefore, increased levels of lysophospholipids would be associated with oxidative stress exposure of depressive subjects. Also, LysoPC, and LysoPE are generated from phosphatidylcholine (PC) and phosphatidylethanolamine (PE), respectively and PC and PE are two major phospholipids that are asymmetrically distributed in the plasma membrane: the majority of PC is localized to the outer leaflet, whereas PE is enriched in the inner leaflet (Devaux, 1991; Jia et al., 2016). The perturbation of LPCs and LPEs might reflect the dysfunction of PC and PE, which might be linked to structural membrane changes and altered permeability of the plasma membrane. Li et al. have reported that liver damage might alter phospholipid levels in the plasma membrane and reduce plasma membrane integrity in a choline-deficient (CD) mouse model (Gruner, 1985; Jia et al., 2016). PBR caused down-regulation of the up-regulated sphingolipids and lyso-glycerophospholipids to a level close to normal in CUMS rats, and this included Cer(d18:0/16:0), Cer(d18:0/18:0), 3-O-Sulfogalactosylceramide (d18:1/22:0), LPE(0:0/18:1(9Z), and LPCs, indicating that PBR has an antidepressant effect through regulating sphingolipid and glycerophospholipid metabolism although it had a comparatively mild adverse effect in CUMS rats. Also, after PBR administration, healthy rats exhibited perturbation of sphingolipids and glycerophospholipids, with down-regulated LPE(0:0/18:1(9Z), LPC(15:0), LPC(20:3), and sphingolipids (except sphinganine 1-phosphate was up-regulated) and up-regulated lysoPCs ]including LPC(16:1), LPC(20:4), LPC(20:0), LPC(17:0)]. PBR might cause liver damage by altering hepatic cell survival and change structural membrane permeability of the plasma membrane in liver tissue of healthy rats, due to the perturbation of sphingolipids and glycerophospholipids which are major constituents of cell membranes. Other than abnormal sphingolipid metabolism and glycerophospholipid metabolisms, an alteration of fatty acid metabolism was also found in CUMS rats, with up-regulated 13-hydroxyoctadecadienoic acid, 10Z-Heptadecenoic acid, N-Heptanoylglycine and L-palmitoylcarnitine and down-regulated octadecenylcarnitine. Changes in fatty acids have also been found in patients with cardiovascular diseases and psychiatric disorders, such as major depression and schizophrenia (Assies et al., 2014). 13s-Hydroxyoctadecadienoic acid is synthesized from linoleic acid by the lipoxygenase pathway and Hennebelle et al. have reported that 13-hydroxyoctadecadienoic acid is increased in the plasma of patients with seasonal major depression in winter (Hennebelle et al., 2017). In a case–control study of 137 patients with recurrent major depression, the concentrations of most of the shorter chain (<or = 18C) saturated fatty acids (SFAs) and monounsaturated fatty acids (MUFAs) were significantly higher (Assies et al., 2010, 2014). Elevated levels of 13-hydroxyoctadecadienoic acid and 10Z-Heptadecenoic acid which is monounsaturated fatty acid (MUFA) in CUMS rats were consistent with those in previous studies (Assies et al., 2010; Hennebelle et al., 2017). N-Heptanoylglycine is an acylglycine with a C-7 fatty acid group as the acyl moiety. Elevated levels of certain acylglycines have been found in the urine and blood of patients with a variety of fatty acid oxidation disorders. In addition, the abnormal acyl carnitines involved in fatty acid metabolism as shown in our study might be associated with depression by disturbing energy metabolism. L-Palmitoylcarnitine and octadecenylcarnitine, involved in oxidation of fatty acids, are a long-chain acyl fatty acid esters of carnitine, and L-palmitoylcarnitine accumulates in the cytosol and serum of patients suffering from mitochondrial carnitine palmitoyltransferase II deficiency. Energy production from long-chain fatty acids (LCFAs) requires transport of LCFAs into the mitochondrial matrix and this transport is carnitine-dependent and involves translocation machinery (Malaguarnera et al., 2011; Ren et al., 2013). Raised levels of L-palmitoylcarnitine and reduced levels of octadecenylcarnitine in plasma of CUMS rats may perhaps lead to disrupted transport of LCFAs into the mitochondrial matrix, subsequently affecting energy production. PBR caused down-regulation of the elevated metabolites in CUMS rats while, in healthy rats, these metabolites remained unaltered following PBR administration except for L-palmitoylcarnitine and octadecenylcarnitine. However, octadecenylcarnitine did not return to normal in CUMS rats after PBR treatment, implying that there might not be regulation on the regulated metabolic site of octadecenylcarnitine in terms of the compatibility of prescribed medicines. In addition, following PBR administration, healthy rats also exhibited perturbed fatty acid metabolism. Acyl carnitines, which included L-palmitoylcarnitine, octadecenylcarnitine and stearoylcarnitine, were significantly altered in healthy rats following PBR administration. Acyl carnitines play important roles in physiological activities and facilitate the transfer of long-chain fatty acids from the cytoplasm to mitochondria during the oxidation of fatty acids, thereby producing energy (as explained earlier) (Malaguarnera et al., 2011; Ren et al., 2013). Also, the study showed that acetyl-L-carnitine treatment can improve liver function and quality of life in patients with minimal hepatic encephalopathy (Malaguarnera et al., 2011). Therefore, PBR might cause mitochondrial dysfunction by altering acyl carnitines, leading to hepatic cell damage and, ultimately, liver damage. Other than altered acyl carnitines, the perturbed lipid peroxidation products of 20-COOH-leukotriene E4, 20-oxo-leukotriene E4 and a-12(13)-EpODE, which were transformed from polyunsaturated fatty acids, were also only found in healthy rats after PBR treatment and not in CUMS rats. 20-COOH-leukotriene E4 and 20-oxo-leukotriene E4, oxidized products of 20-COOH-leukotriene, are metabolites obtained by lipid oxidation of leukotriene E4. Leukotrienes are oxylipins synthetized from polyunsaturated fatty acids via the lipoxygenase (LOX) pathway (Hennebelle et al., 2017) and leukotriene E4 is a potent inflammatory mediator (Assies et al., 2014). Alpha-12(13)-EpODE [a-12(13) epoxyoctadecadienoic acid], an oxygenated lipid, is also produced by the oxidation of linoleic acid via the CYP450 pathway (Hennebelle et al., 2017). Increased oxidative stress results in peroxidation of the cell membrane and plasma lipids which are considered primary targets of oxidative stress (Vural et al., 2017). Therefore, elevated levels of lipid peroxidation products of 20-COOH-leukotriene E4 and a-12(13)-EpODE might be associated with the effect of oxidative stress on polyunsaturated fatty acids. PBR might cause liver damage by accumulation of lipid peroxidation products and triggering inflammatory reactions. Among the metabolites involved in the three pathways of sphingolipid metabolism, glycerophospholipid metabolism and fatty acid metabolism, the seven metabolites of sphinganine, Cer(d18:0/16:0), Cer(d18:0/18:0), LPC(15:0), LPC (20:3), LPE(0:0/18:1(9Z), and palmitoylcarnitine were the same regulative differential metabolites and showed the same down-regulation in both CUMS rats and healthy rats following PBR treatment while, in CUMS rats, these metabolites could return to normal following PBR administration, except that the relative content of palmitoylcarnitine became less than normal at high doses. This suggests that PBR has a comparatively mild adverse effect in CUMS rats. Sphingolipid, glycerophospholipid, and fatty acid metabolism were identical regulated pathways in both CUMS rats and healthy rats following PBR administration, but some regulated target sites of metabolites of PBR were different, suggesting that PBR affected different sites of action in the three pathways under different physiological conditions.

Perturbed differential metabolites and altered metabolic pathways in healthy rats after exposure to PBR could well explain the toxic effect of the drug. Elevated levels of 2-Keto-6-acetamidocaproate and (S)-2-amino-6-oxohexanoate were observed only in healthy rats after PBR treatment but not in CUMS rats. 2-Keto-6-acetamidocaproate is an intermediate in lysine degradation and can be further converted to (S)-2-amino-6-oxohexanoate. It can be generated from N6-acety-L-lysine which is an acetylated amino acid. Lysine acetylation, which is a reversible post-translational modification of lysine residues in various proteins, has recently been identified as a novel regulator of mitochondrial bioenergetic function and controls numerous cellular processes (Anderson and Hirschey, 2012; Thapa et al., 2017). Increased levels of 2-Keto-6-acetamidocaproate and (S)-2-amino-6-oxohexanoate as shown in our study in healthy rats given PBR shows that lysine acetylation could be accelerated and this might further disturb mitochondrial and cellular functions and ultimately caused hepatic cell damage. Perturbation of sphingolipids and glycerophospholipids are implicated in healthy rats following PBR administration and PBR might cause liver damage by altering hepatic cell survival and structural plasma membrane permeability in liver tissue (as explained earlier). In addition, PBR might also cause liver damage by accumulating lipid peroxidation products and triggering an inflammatory reaction, and cause mitochondrial dysfunction by altering acyl carnitines and producing hepatic cell damage and, ultimately, cause liver damage (as explained earlier). Moreover, bile acid metabolism was significantly altered only in healthy rats following PBR treatment, but was not altered in CUMS rats. Bile acids, the major metabolites of cholesterol in hepatic cells, play an important physiological role in the elimination of cholesterol from the body (Burkard et al., 2005). In hepatobiliary diseases, the elimination of total bile acids was inhibited, giving rise to an increased concentration of total bile acids in peripheral blood (Burkard et al., 2005). Abnormally high concentrations of bile acids, such as can occur in cholestasis, can result in intrahepatic accumulation of toxic bile acids leading to hepatic damage by producing pathophysiological effects including mitochondrial dysfunction with overgeneration of reactive oxygen and nitrogen species(Jaeschke et al., 2002; Palmeira and Rolo, 2004; Tan et al., 2007; Tian et al., 2017). Moreover, even minor liver damage can cause perturbation of serum and hepatic bile acids (Yamazaki et al., 2013; Tian et al., 2017). Various liver disorders, such as nonalcoholic fatty liver disease (NAFLD), and drug-induced liver injury can increase the levels of bile acids in liver (Lake et al., 2013; Tian et al., 2017). Therefore, bile acids are considered as highly sensitive markers of liver injury and liver dysfunction, and are used as potential biomarkers in drug-induced liver injury. Therefore, PBR might cause liver injury which is associated with, and potentially linked to the increased bile acid concentrations in this study. Taken together, the facilitation of lysine degradation and the disruption of sphingolipid metabolism, glycerophospholipid metabolism and oxidation of fatty acids as well as bile acid metabolism by PBR could be partly responsible for its toxic effects in healthy rats. However, the improvement in depression of PBR shown in our study due to its regulation of glycometabolism, amino acid metabolism, sphingolipid, glycerophospholipid and fatty acid metabolism could support the use of PBR as an antidepressant in subjects with depression.

Our results have demonstrated that PBR has an antidepressant effect through regulating glycometabolism, amino acid metabolism, sphingolipid metabolism, glycerophospholipid metabolism and fatty acid metabolism, while it can produce more severe toxic effects in the liver or kidney in healthy rats compared with CUMS rats. Glycometabolism and bile acid metabolism were the most significant of the regulated pathways of PBR in the different physiological states. Also, amino acid metabolism, sphingolipid metabolism, glycerophospholipid metabolism and fatty acid metabolism were the identical regulated pathways in both different physiological states of depression and normal, while some regulated target sites for metabolites of PBR were different. These results suggest that PBR has an influence on different sites of action in different physiological conditions. This phenomenon, that PBR can cause hepatic and renal damage in healthy rats but has a comparatively mild adverse effect in CUMS rats, supports the traditional Chinese medicine (TCM) theory of “You Gu Wu Yun” (translated as a toxic herb would not produce toxicity in a corresponding pathological state, on the contrary, it would have a therapeutic effect). This theory has been regarded as one of the most important guiding principles in TCM clinical practices for centuries (Tan et al., 2013). It had been supported by deciphering the differential toxic response of *Radix aconiti lateralis praeparata* in healthy and hydrocortisone-pretreated rats (Tan et al., 2013). In addition, it is worth noting that whether some enzymes activities, such as aSMase and phospholipases A2, are affected by PBR there is a need for further investigations. The results in this study suggest that the guidance of the theory of TCM syndrome differentiation plays an important role in the use of Chinese medicines, and might also indicate that the compatibility and processing of Chinese herbs is necessary and of scientific importance in reducing the potential toxicity of traditional Chinese medicines.

## Conclusions

In the current study, the differential effects and toxic responses of Bupleuri Radix in pathological and physiological responses in the organism were investigated. The results obtained demonstrated that PBR exhibits an antidepressant effect by regulating glycometabolism, amino acid metabolism, sphingolipid, glycerophospholipid, and fatty acid metabolism in CUMS rats. However, PBR can produce more severe toxic reactions in the liver or kidney in healthy rats than in CUMS rats by facilitation of lysine degradation and disruption of sphingolipid, glycerophospholipid and oxidation of fatty acid as well as the bile acid metabolism. PBR affects different sites of action in different physiological conditions. The differential effective and toxic response of PBR in CUMS rats and healthy rats provide a new reference for assessing the more rational and safer application of clinical drugs in the future.

## Author contributions

XG, ML, and YF conceived and designed the experiments; ML and XG wrote the paper; ML, YF, and FZ performed the experiments; ML and YF analyzed the data; JT and XQ. design of the study and writing the protocol; XZ helpful revision on the text and grammar. All authors have given approval to the final version of the manuscript.

### Conflict of interest statement

The authors declare that the research was conducted in the absence of any commercial or financial relationships that could be construed as a potential conflict of interest.
