# Supplementary material for: Deciphering the Differential Effective and Toxic Responses of Bupleuri Radix following the Induction of Chronic Unpredictable Mild Stress and in Healthy Rats Based on Serum Metabolic Profiles
Source: Front Pharmacol. 2018 Jan 15;8:995. doi: 10.3389/fphar.2017.00995 (PMC5775221; doi:10.3389/fphar.2017.00995)
Supplement: Supplementary file 1 [file DataSheet1.DOCX]

**Deciphering the Differential Effective and Toxic Responses of Bupleuri Radix following the induction of Chronic Unpredictable Mild Stress and in Healthy Rats Based on Serum Metabolic Profiles**

Xiaoxia Gao^a, c, △, *^, Meili Liang^a, b, △^, Yuan Fang^a^, Fang Zhao^a^, Junsheng Tian^a, c^, Xiang Zhang^a, d^, Xuemei Qin^a, c, *^

^a^ Modern Research Center for Traditional Chinese Medicine, Shanxi University, Taiyuan 030006, China

^b^ College of Chemistry and Chemical Engineering, Shanxi University, Taiyuan 030006, China

^c^ Key Laboratory of Chemical Biology and Molecular Engineering of Ministry Education of Shanxi University, Taiyuan 030006, China

^d^ University Of Louisville, Louisville, 40292, USA

**AUTHOR INFORMATION**

**Corresponding Author**

^*^Xiaoxia Gao, Phone: 86-351-7019297, Fax: 86-351-7011202, E-mail: [gaoxiaoxia@sxu.edu.cn;](mailto:gaoxiaoxia@sxu.edu.cn;)

Xuemei Qin, Phone: 86-351-7011501, Fax: 86-351-7011202, E-mail: [qinxm@sxu.edu.cn;](mailto:qinxm@sxu.edu.cn;)

**Author Contributions**

^△^These authors contributed equally to this work. X.X.G., M.L.L., Y.F., conceived and designed the experiments; M.L.L and X.X.G wrote the paper; M.L.L., Y.F., F.Z. performed the experiments; M.L.L., Y.F. analyzed the data; J.S.T., X.M.Q. design of the study and writing the protocol; X.Z. helpful revision on the text and grammar. All authors have given approval to the final version of the manuscript.

A

[K] group

[CM] group

[C4] group

[C6] group

[C7] group

B

[K] group

[CM] group

[C4] group

[C6] group

[C7] group

C

[K] group

[Z4] group

[Z6] group

[Z7] group

[QC] group

D

[K] group

[Z4] group

[Z6] group

[Z7] group

[QC] group

Figures S1. (A) UPLC/MS total ion chromatograms of serum samples in positive ion mode of [K] group, [CM] group, [C4] group, [C6] group, [C7] group. (B) UPLC/MS total ion chromatograms of serum samples in negative ion mode of [K] group, [CM] group, [C4] group, [C6] group, [C7] group. (C) UPLC/MS total ion chromatograms of serum samples in positive ion mode of [K] group, [Z4] group, [Z6] group, [Z7] group and [QC] group. (D) UPLC/MS total ion chromatograms of serum samples in negative mode of [K] group, [Z4] group, [Z6] group, [Z7] group and [QC] group.

Table S1. The stability of UHPLC -Q Exactive Orbitrap -MS method using QC sample.

| NO. | RT (min) | RSD (%) | m/z | RSD (%) | ion |
| --- | --- | --- | --- | --- | --- |
| 1 | 1.18 | 0.7913 | 146.0812 | 1.53×10^-4^ | M+H |
| 2 | 1.38 | 0.6685 | 146.0453 | 3.41×10^-5^ | M-H |
| 3 | 3.22 | 1.086 | 132.1025 | 1.4×10^-5^ | M+H |
| 4 | 5.99 | 0.1992 | 205.0976 | 2.39×10^-5^ | M+H |
| 5 | 9.71 | 0.1066 | 190.0868 | 2.27×10^-5^ | M+H |
| 6 | 18.02 | 0.2902 | 302.3058 | 3.11×10^-5^ | M+H |
| 7 | 22.08 | 0.3419 | 400.3426 | 1.65×10^-5^ | M+H |
| 8 | 23.82 | 0.0923 | 862.5595 | 7.71×10^-5^ | M-H |
| 9 | 25.13 | 0.1122 | 496.3402 | 2.5×10^-5^ | M+H |
| 10 | 28.36 | 0.0373 | 482.3245 | 4.0×10^-5^ | M+H |
